# Cognitive control in media multitaskers: Two replication studies and a meta-Analysis

**DOI:** 10.3758/s13414-017-1408-4

**Published:** 2017-08-24

**Authors:** Wisnu Wiradhany, Mark R. Nieuwenstein

**Affiliations:** Department of Experimental Psychology, Research School of Behavioural and Cognitive Neurosciences, University of Groningen, Grote Kruistraat 2/1, 9712 TS Groningen, The Netherlands

**Keywords:** Media multitasking, Distractibility, Selective attention, Working memory, Task switching

## Abstract

Ophir, Nass, and Wagner (2009, *Proceedings of the National Academy of Sciences of the United States of America*, *106*(37), 15583–15587) found that people with high scores on the media-use questionnaire—a questionnaire that measures the proportion of media-usage time during which one uses more than one medium at the same time—show impaired performance on various tests of distractor filtering. Subsequent studies, however, did not all show this association between media multitasking and distractibility, thus casting doubt on the reliability of the initial findings. Here, we report the results of two replication studies and a meta-analysis that included the results from all published studies into the relationship between distractor filtering and media multitasking. Our replication studies included a total of 14 tests that had an average replication power of 0.81. Of these 14 tests, only five yielded a statistically significant effect in the direction of increased distractibility for people with higher scores on the media-use questionnaire, and only two of these effects held in a more conservative Bayesian analysis. Supplementing these outcomes, our meta-analysis on a total of 39 effect sizes yielded a weak but significant association between media multitasking and distractibility that turned nonsignificant after correction for small-study effects. Taken together, these findings lead us to question the existence of an association between media multitasking and distractibility in laboratory tasks of information processing.

**Electronic supplementary material:**

The online version of this article (10.3758/s13414-017-1408-4) contains supplementary material, which is available to authorized users.

Over the past two decades, the amount of information that is available online through the World Wide Web has increased exponentially (Palfrey & Gasser, 2008), and the accessibility of this information has likewise increased with the introduction of various modern multimedia devices (e.g., Lenhart, 2015). Taken together, these developments have led to two major changes in individual behavior. First, people spend many hours per day online, as indicated by a recent survey from Pew research center, which showed that 24% of teens in the United States report being online “almost constantly” (Lenhart, 2015). Second, people tend to engage in media multitasking (e.g., Brasel & Gips, 2011; Judd & Kennedy, 2011): Instead of being focused on a single task or stream of information, they try to monitor and interact with multiple streams of information simultaneously.

The fact that many people nowadays spend large portions of their waking lives in a media-rich environment raises the interesting question as to whether this experience might influence the information-processing mechanisms of the mind and brain. That is, could the frequent engagement in media multitasking have benefits for our ability to deal with multiple streams of information? In a recent study, Ophir, Nass, and Wagner (2009) addressed this question, and their results produced a surprising conclusion. In the study, Ophir and colleagues introduced the media-use questionnaire as a measure of the proportion of media-usage time during which people consume more than one type of media, and they used the resulting Media Multitasking Index (MMI) to conduct a quasi-experimental study in which the performance of participants with a high and low MMI was compared for several widely used measures of information processing (see Table 1).

**Table 1:** Tasks, analyses, and effects reported by Ophir et al. (2009)

Task	Conditions included	Findings and effect sizes in Ophir et al. (2009)	P(rep) Exp. 1	P(rep) Exp. 2
Change detection	Memory set of 2, with 0, 2, 4, or 6 distractors	Interaction of group (LMM vs. HMM) and number of distractors for Memory Set Size 2 condition (*f* = .34; *d =* .68): HMMs showed a decline in performance with increasing numbers of distractors; LMMs did not	.95	.97
	Memory set of 4, with 0, 2, or 4 distractors			
	Memory set of 6, with 0 or 2 distractors	*No analyses reported for conditions with 4 and 6 targets*		
	Memory set of 8, with 0 distractors	No significant difference in memory capacity of HMMs and LMMs in comparison of memory sets of 2, 4, 6, and 8 items, without distractors		
AX-CPT	With vs. without distractors	Significant interaction of group (LMM vs. HMM) and distractors (present vs. absent) for response times: HMMs slower to respond to target (*d* = 1.19) and nontarget (*d* = 1.19) probes only in the condition with distractors	.86	.76
			.86	.76
*N*-back task	2-back vs. 3-back	Interaction of Group (LMM vs. HMM) × Condition (2-back vs. 3-back) for false-alarm rate, with HMMs showing a stronger increase in false alarms as memory load increased from 2-back to 3-back (*f* = .42; *d =* .84)	.95	.92
Task switching: number-letter	Task-repeat and task-switch trials	HMMs showed significantly slower response times for both switch (*d* = .97) and repeat (*d* = .83) trials and a larger switch cost (*d* = .96)	.72	.80
			.60	.69
			.71	.79
Stop-signal task	*Not specified*	No analyses reported, but Ophir et al. did mention there was no significant difference between LMMs and HMMs		
Stroop task	*Not specified*	*No analyses reported*		
Task switching	*Not specified*	*No analyses reported*		

*Note.* LMM = light media multitaskers; HMM = heavy media multitaskers; *d =* effect size in Cohen’s *d* for the effects reported by Ophir et al.; P(rep) = acquired replication power for our replication tests with α = .05

Specifically, as can be seen in Table 1, the participants in Ophir et al.’s study completed two task-switching experiments, a change-detection task with and without distractors, an *N*-back task with two levels of memory load (two-back and three-back), an AX-continuous-performance task (AX-CPT) with and without distractors, a Stroop task, and a stop-signal task. Surprisingly, the results showed that people with high scores on the media-use questionnaire were impaired when the task required some form of filtering out irrelevant, distracting information, such that heavy media multitaskers (HMMs)—but not light media multitaskers (LMMs)—were negatively affected by the presence of distractors in the change-detection and AX-CPT tasks. In addition, the results showed that HMMs made more false alarms in the *N*-back task, and they showed slower response times and larger switch costs in the task-switching experiment. In interpreting these findings, Ophir et al. argued that HMMs had difficulty in suppressing the memory representations of earlier encountered targets in the *N*-back task, and that they had difficulty in inhibiting a previously used task set in the task-switching experiment. Accordingly, Ophir et al. concluded that “heavy media multitaskers are more susceptible to interference from irrelevant environmental stimuli and from irrelevant representations in memory” (p. 15583).

## Results of follow-up studies to Ophir et al.’s (2009) pioneering work

Following Ophir et al.’s (2009) pioneering study, several reports were published that followed up on this pioneering work by examining the association between questionnaire measures of media multitasking and various measures of information-processing capacity, distractibility, brain functioning, personality, and daily-life functioning. The results of these studies present a large and mixed set of results.

On the one hand, some studies found correlates of the MMI with lower working-memory capacity (Cain, Leonard, Gabrieli, & Finn, 2016; Sanbonmatsu, Strayer, Medeiros-Ward, & Watson, 2013), limited top-down control over visual selective attention (Cain & Mitroff, 2011), lower gray-matter density in the anterior cingulate cortex (Loh, Kanai & Watanabe, 2014), lower scores on measures of fluid intelligence (Minear, Brasher, McCurdy, Lewis, & Younggren, 2013), an improved ability for dividing spatial attention (Yap & Lim, 2013) an improved ability to integrate visual and auditory information (Lui & Wong, 2012), more frequent self-reports of depression and social anxiety symptoms (Becker, Alzahabi, & Hopwood, 2013), higher scores on certain subscales of self-report measures of impulsivity (Minear et al., 2013; Sanbonmatsu et al., 2013), increased self-reports of attentional lapses and mind-wandering in daily life (Ralph, Thomson, Cheyne, & Smilek, 2013), lower academic achievement (Cain et al., 2016), and with lower self-reports for executive functioning in daily life (Baumgartner, Weeda, van der Heijden, & Huizinga, 2014). At the same time, however, these studies also reported nonsignificant associations for various other outcome measures, and the results of studies that examined the association between MMI and outcome measures similar to those used by Ophir et al. generally failed to replicate the original effects. For instance, Baumgartner et al. (2014) found that participants with higher scores for media multitasking were less, not more, susceptible to distraction in the Eriksen flanker task, and Ophir et al.’s original finding of an association with increased susceptibility to distraction in a change-detection task was also not replicated in several other studies (Cardoso-Leite et al., 2015; Gorman & Green, 2016; Uncapher, Thieu, & Wagner, 2015). Likewise, Ophir et al.’s finding of increased switch costs in HMMs was not replicated in four subsequent studies (Baumgartner et al., 2014; Cardoso-Leite et al., 2015; Gorman & Green, 2016; Minear et al., 2013), with one study showing that HMMs had less, not more, difficulty in switching tasks than LMMs (Alzahabi & Becker, 2013).

## The current study

Taken together, it can be concluded that while the follow-up studies to Ophir et al.’s (2009) pioneering study reported evidence suggestive of various correlates of media multitasking, the original findings by Ophir et al. were not always replicated. Thus, it can be said that the currently available evidence regarding a relationship between media multitasking and distractibility is mixed and in need of further scrutiny. To shed further light on the possible existence of this relationship, we conducted two replication studies that included all experiments that showed a deficit in HMMs in the original study by Ophir et al., and we conducted a meta-analysis that included the results of all studies probing the existence of a relationship between media multitasking and distractibility in laboratory tasks of information processing. While the replication studies were done to afford insight into the replicability of Ophir et al.’s specific findings, the meta-analysis was conducted to provide a test of the strength of the relationship media multitasking and distractibility across all studies done to date.

### Justification of methods and approach to statistical inference

In this section, we will describe and motivate our approach in testing the existence of a relationship between media multitasking and distractibility. As alluded to above, this approach involved the use of replication tests for the specific findings of Ophir et al. (2009; see Table 1) and involved the use of a meta-analysis to quantify the strength of the MMI–distractibility link across all studies that have probed this relationship, including the two replication studies reported here. While the outcomes of our replication studies shed light on the replicability of the specific effects found by Ophir et al., the meta-analysis can provide an answer to the more central question of whether there exists an association between media multitasking and distractibility in general, and for certain types of tasks in particular. Our choice for relying on the meta-analysis for an answer to the main question of whether there exists an association between media multitasking and distractibility was motivated by the fact that this association has been examined in several other studies, and that, therefore, the most powerful, reliable answer to this question can be gained from considering the evidence that all of these studies provide together.

For the replication studies, we adhered to the recommendations provided for replication research (e.g., Brandt et al., 2014; Open Science Collaboration, 2015). To start, we carefully identified the main findings of interest reported by Ophir et al. (2009) and selected them as our targets for the replication tests.^1^ Secondly, we copied the methods of Ophir et al. as closely as possible to ensure there were no methodological differences that could explain any differences in outcomes. Thirdly, we aimed to include as many participants as possible to ensure a reasonable level of power for successful replication of Ophir et al.’s results, if they were real. Fourthly, we adhere to the recommendations provided by the Psychonomic Society in that we used a rigorous set of statistical methods to evaluate the outcomes of our replication studies. In the following sections, we will further elaborate on how these four points were implemented in our replication studies.

#### Selection of outcomes of interest for replication studies

For the replication tests, a first point of consideration was that the study by Ophir et al. (2009) included several tasks that had different conditions and different outcomes (e.g., accuracy and response times for four types of trials in the AX-CPT), which were in some cases examined in several different analyses. To avoid the risk of inflation of null-hypothesis rejection rates with multiple testing, a first step in our replication efforts was to select the main findings of interest from Ophir et al. In doing so, we closely examined the report of Ophir et al. to determine which findings were used as the basis for their conclusion that there exists an association between media multitasking and increased distractibility. Our analysis of this matter identified seven key findings (see Table 1), and these findings thus became our outcomes of interest in examining the replicability of Ophir et al.’s findings. Specifically, for the change-detection task, Ophir et al. reported a significant group by distractor set size interaction for the condition with two targets. For the AX-CPT, the main finding of interest was that HMMs showed slower responses in the condition with distractors, but only on trials in which the probe required participants to refer to the cue they had to maintain in memory during the presentation of the distractors separating the cue and the probe (AX and BX trials). For the *N*-back task, this was the finding of an interaction between group and working-memory load for false alarms, such that HMMs showed a stronger increase in false alarms as load increased across the two-back and three-back conditions. Lastly, for the task-switching experiment, Ophir et al. found that HMMs were slower on both switch and nonswitch trials, and they also showed a larger switch cost (i.e., a larger difference in response times for switch and nonswitch trials). In discussing these three results, Ophir et al. took each to reflect evidence for increased distractibility (cf. description of results on p. 15585 in Ophir et al.), and, accordingly, we selected each of these three outcomes of the task-switching experiment as targets for our replication attempt.

#### Methods used in the replication studies

For our replication studies, we aimed to replicate the methods of Ophir et al. (2009) as closely as possible. Specifically, we first asked as many participants as possible to fill in the same media-use questionnaire that was used by Ophir et al., and we then assigned participants with scores in the first quartile of the distribution of media multitasking scores to the LMM group, whereas participants with scores in the fourth quartile were assigned to the HMM group. These participants were invited to take part in a lab study. In using the same group of participants for all experiments in the lab study, our procedure differed from that of Ophir et al. because Ophir et al. used different groups of participants for different tasks. In addition, our procedure differed from that of Ophir et al. because we used quartiles as the criteria for the assignment of participants to the LMM and HMM groups, whereas Ophir et al. assigned participants to these groups on the basis of their scores being one standard deviation below or above the group mean. Our choice for using quartiles, as opposed to using Ophir et al.’s standard-deviation-based criterion, was motivated by practical and empirical considerations as the use of quartiles would result in larger groups of participants in the LMM and HMM groups, and, furthermore, some previous studies have been successful in identifying differences between LMMs and HMMs using the quartile-based approach (Cain & Mitroff, 2011; Yap & Lim, 2013).

To ensure that the methods we used for the experiments in the lab study were identical to those used by Ophir et al. (2009), we requested and received the original experiment programs used by Ophir et al. This allowed us to copy the exact methods of Ophir et al. for our replication studies. However, there was one task for which we did not copy Ophir et al.’s methods exactly. This concerned the AX-CPT, for which we chose not to include a condition without distractors, since Ophir et al. found that HMMs only performed worse than LMMs when this task was done in the presence of distractors. Except for the omission of this condition without distractors, the AX-CPT was identical to the task used by Ophir et al., and the other tasks—change detection, *N*-back, and task-switching—were all identical to those used by Ophir et al. as well.

#### Data analysis for the replication studies

In analyzing the results of our replication attempts, we complied with the statistical guidelines of the Psychonomic Society (Psychonomic Society, 2012). As stated in these guidelines, the conventional approach of null-hypothesis significance testing (NHST) has several vulnerabilities, and researchers should therefore be encouraged to supplement the results of NHSTs with other metrics and analyses, such as power analyses, effect sizes and confidence intervals, and Bayesian analyses. In implementing this recommendation, we first computed our acquired replication power to determine the likelihood that we would be able to replicate the effects of interest, given our sample size. As detailed below, these power analyses showed that our sample sizes were sufficiently large to yield an average replication power of .81, which is generally considered to be an acceptable level of power (Cohen, 1992). To determine whether our replication attempts were successful, we conducted NHSTs to determine whether the effects of interest reached significance at α = .05, and, in doing so, we used one-sided tests for directional predictions that could be tested using a *t* test. For hypotheses involving more than two condition means, we reported the regular *F* statistics, as these are one-sided by definition. In interpreting the results of these NHSTs, we refrained from interpreting nonsignificant results with *p* < .1 as trends, as it has been demonstrated that such nonsignificant results should not be taken to reflect a trend in the direction of statistical significance, because the inclusion of additional data will not necessarily result in a lower *p* -value (Wood, Freemantle, King, & Nazareth, 2014). In addition to conducting the NHSTs, we also calculated effect sizes and their confidence intervals to gain further insight into the strength of both significant and nonsignificant effects. Lastly, we also conducted a Bayes factors analysis. As detailed below, this type of analysis is an important supplement to NHST because it provides a more conservative estimate of the extent to which the data support the presence of an effect, and because it also allows one to determine the extent to which a nonsignificant result provides evidence in favor of the null hypothesis.

#### Bayes factors analyses

As alluded to above, a Bayes factors analysis allows one to quantify the extent to which the acquired data support the existence (*H*_*1*_) or absence (*H*_*0*_) of an effect, with a continuous measure that expresses the ratio of the likelihood of the data under these respective hypotheses (Jarosz & Wiley, 2014; Rouder, Morey, Speckman, & Province, 2012; Rouder, Speckman, Sun, Morey, & Iverson, 2009; Wagenmakers, 2007). This measure has advantages over the traditional approach of significance testing because it allows for an assessment of the evidence for both *H*_*1*_ and *H*_*0*_, instead of only allowing the rejection of *H*_*0*_ if the observed data is unlikely under the null hypothesis (i.e., less than α). Furthermore, it has been shown that, compared to significance tests, Bayes factors provide a more robust test of the acquired evidence because significance tests tend to overestimate the evidence against *H*_*0*_. Specifically, when adopting a *BF*_*10*_ > 3 as the criterion for the presence of an effect, it has been found that 70% of 855 effects that reached significance with *p* values between .01 and .05 did not reach this threshold of *BF*_*10*_ > 3 (Wetzels et al., 2011). Thus, a Bayes factors analysis not only supplements the NHST in allowing for a quantification of evidence in favor the null hypothesis but it can also be said to provide a more conservative test for the presence of an effect than that provided by NHST.

In calculating Bayes factors, we assumed the default prior values included in BayesFactor package in R (Morey, Rouder, & Jamil, 2015), and we expressed the evidence in terms of *BF*_*01*_ (ratio of likelihood of data given *H*_*0*_ : likelihood of data given *H*_*1*_) in case our significance test yielded a nonsignificant effect, and in terms of *BF*_*10*_ (ratio of likelihood of data given *H*_*1*_ : likelihood of data given *H*_*0*_) in case the significance test yielded a statistically significant effect. For all *BF*s, values greater than one signified evidence in favor of one hypothesis over the other, with greater values signifying greater evidence. In characterizing the resulting *BF*s, we followed the nomenclature of Jeffreys (1961), which considers *BF*s of 1–3 as anecdotal evidence, 3–10 as moderate evidence, 10–30 as strong evidence, and 30–100 as very strong evidence.

## Experiment 1

### Method

#### Participants

A total of 154 undergraduate students from the Faculty of Psychology, Universitas Gadjah Mada, Indonesia, were invited to fill in the media-use questionnaire in an online study. Of these 154 participants, 148 participants completed the questionnaire. The MMI scores were normally distributed, as indicated by a Kolmogorov–Smirnov test, *Z* = .70, *p* = .49, with an average score of 6.80 and a standard deviation of 1.98. Using the lower and upper quartiles of the distribution of MMI scores as criteria, we classified 23 participants as LMMs and 24 as HMMs. These participants were invited for a lab study for which they would receive a monetary compensation of 50.000 rupiah (~3.5 €). In total, 13 HMMs (*M*_MMI_ = 9.74, *SD* = .66) and 10 LMMs (*M*_MMI_ = 4.09, *SD* = 1.12) responded to our invitation for the lab study.

#### Materials and general procedure

The materials used for the replication studies included the same media-use questionnaire as that used by Ophir et al. (2009) and four experiments (change detection, *N*-back, AX-CPT, and task switching), which showed the main effects of interest (see Table 1). As in Ophir et al. (2009), the questionnaire was set out in an online study. The data for the four experiments were collected in an open computer lab equipped with multiple Intel i3 desktop computers, which had a 2.6 GHz CPU and 2 GB of RAM. Stimuli were presented on a 20-inch LCD monitor, and the presentation of stimuli and collection of responses were controlled using software written in PsychoPy Version 1.8.2. (Peirce, 2007). The responses were recorded using a QWERTY keyboard. Each of the four tasks took approximately 15 minutes to be completed, and the order of the tasks was randomized across participants.

#### The media-use questionnaire

To assess media multitasking, we used the same questionnaire as the one introduced by Ophir et al. (2009). This questionnaire consists of 144 items that each ask the participant the following: When using [one of 12 possible media], how often do you also use [the same media or one of the other 11 media]? The types of media covered by the questionnaire include printed media, e-mail, television, video, music, nonmusic audio, phone, text messaging, instant messaging (e.g., chat), browsing, video games, Internet browser, and other media. To answer the items, the participant is asked to choose between *never*, *sometimes*, *often*, and *almost always*. By combining all 12 types of media, thus including the possibility of using the same medium twice, this yields a total of 144 combinations for which responses are weighted with a value of 0 (*never*), .33 (*sometimes*), .67 (*often*) or 1 (*almost always*). To compute the Media Multitasking Index (MMI), the scores for the 144 items are subsequently entered into the following equation:

$$ MMI=\sum_{i=1}^{12}\frac{m_i\times {h}_i}{h_{total}}, $$

in which *m*_*i*_ is the sum score for media multitasking using primary medium *i, h*_*i*_ is the number of hours spent consuming primary medium *i* per week, and *h*_*total*_ is the sum of hours spent consuming any of the 12 media. The MMI thus indicates the percentage of media-usage time during which a participant uses two media at the same time. Note that by implication, the MMI is insensitive to the actual amount of time people spent using different media at the same time, as the calculation of the MMI entails that 1 hour of media multitasking per day produces the same MMI as 16 hours of media multitasking. This aspect of the MMI has been pointed out in previous studies (Cain et al., 2016; Moisala et al., 2016), and we return to its implications in the general discussion section.

#### Materials, design, and procedure for change detection

**Fig. 1:**
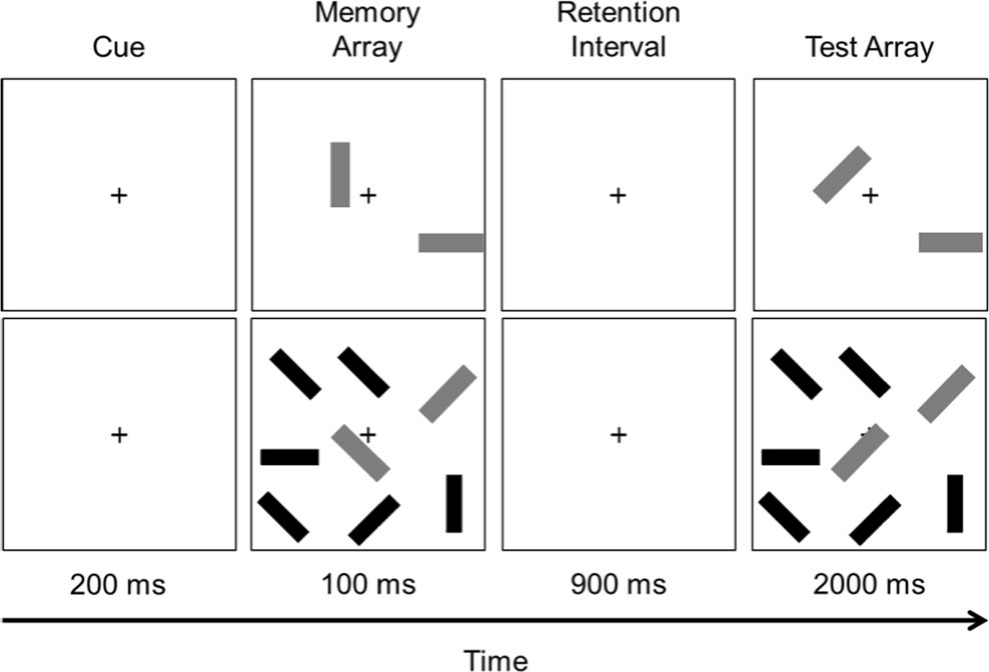
Change detection task with zero distractors (*lower quadrants*) or with six distractors (*upper quadrants*). The examples shown had a memory set size of two items. The *gray* and *black bars* were presented in red and blue, respectively

We used a change-detection task identical to the one used by Ophir et al. (2009), who used a task designed by Vogel, McCollough, and Machizawa (2005). As indicated in Fig. 1, each trial began with the appearance of a fixation cross for 200 ms, which was followed by a 100-ms display of a memory array consisting of two, four, six, or eight red bars that had to be remembered. Except for the memory array with eight red bars, the other arrays could also include blue bars that served as distractors, with the possible numbers of blue bars being [0, 2, 4, or 6], [0, 2, or 4], and [0 or 2], for memory arrays with two, four, and six target elements, respectively. Following the appearance of this array, there was a 900-ms retention interval followed in turn by a test array that was shown for 2,000 ms. In the test array, one of red bars could have a different orientation compared to the same bar in the memory array, and the task for the participants was to press one of two designated keys to indicate whether a red bar had changed its orientation, which was the case on 50% of the trials. Following this response, the test array disappeared, and the memory array for the next trial appeared after 200 ms. The task consisted of a total of 200 trials, yielding 10 change and 10 no-change trials for each combination of memory set size and distractor set size.

#### Materials, design, and procedure for AX-CPT

**Fig. 2:**
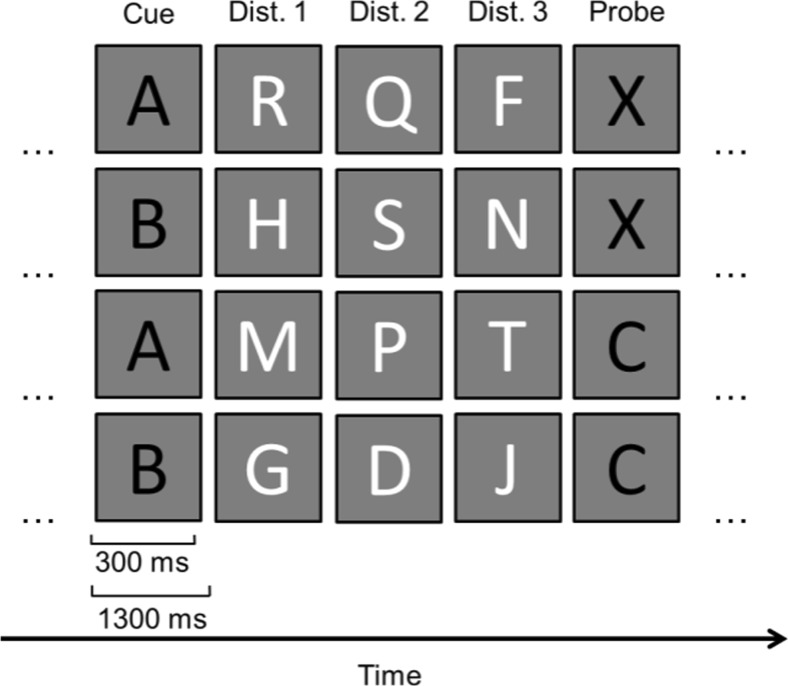
AX-CPT with distractors. The figure shows examples of the subsequences of five letters in the AX, BX, AY, and BY conditions. The *black letters* were presented in red

For the AX-CPT, we used the same task Ophir et al. (2009) used, but we chose to exclude the condition without distractors because Ophir et al. found that HMMs only performed worse than LMMs in the condition with distractors. In the task, participants were shown a continuous sequence of letters that each appeared for 300 ms, followed by a blank interstimulus interval (ISI) of 1,000 ms (see Fig. 2). The sequence was composed of subsequences of five letters, of which the first and last were shown in red, and the task for the participant was to respond with one of two keys on a keyboard to each letter—they had to press the “4” key when they detected a red *X* that was preceded by a red *A*, whereas they had to press the “5” key for all other letters in the sequence (i.e., any other red or white letter). Thus, the task for the participant was to monitor the stream for the occurrence of a red *A* followed in time by the appearance of a red *X*. Across trials, the red letters were selected in such a way that 70% of the subsequences included a red *A* followed by a red *X*, whereas the remaining 30% of the subsequences consisted of trials in which a red *A* was followed by a red letter different than *X* (hereafter denoted the AY trials), or wherein a red letter different than *A* was followed by a red *X* (hereafter denoted BX trials), or wherein a red letter different than *A* was followed by a red letter different than *X* (hereafter denoted BY trials). The experiment consisted of five series of 30 subsequences, and participants were allowed to take a short break after each series.

#### Materials, design, and procedure for *N*-back task

**Fig. 3:**
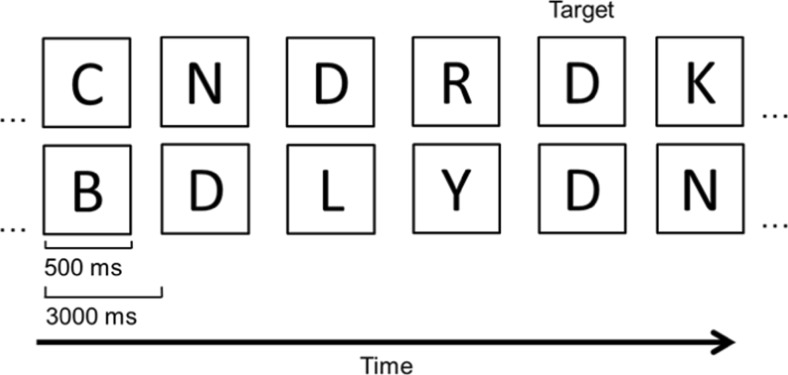
Example of a sequence of letters for the two-back (*top row*) and three-back (bottom row) conditions in the *N*-back task

The *N*-back task was also identical to the task used by Ophir et al. (2009). Participants were presented a sequence of black letters on a white screen. Each letter appeared for 500 ms, followed by a blank ISI for 3,000 ms (see Fig. 3). The task for the participant was to determine if a currently shown letter was the same as the one shown two positions earlier (two-back condition), or three positions earlier (three-back condition). To respond to such targets, participants pressed the “4” key of the keyboard whereas they pressed the “5” key in response to all other letters. The two- and three-back conditions each consisted of the presentation of 90 letters, of which 13 were targets. As in the study by Ophir et al., the two-back condition was always done first, followed in time by the three-back condition.

#### Materials, design, and procedure for task switching

**Fig. 4:**
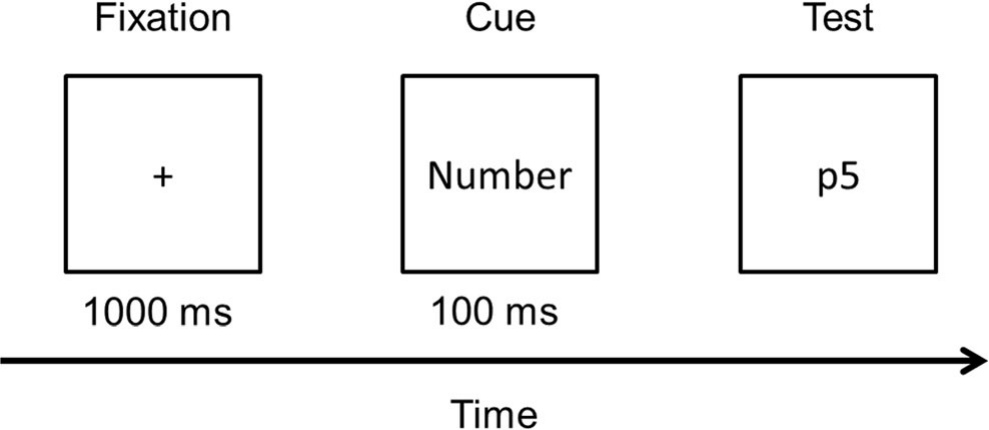
Example of a trial sequence in the number–letter task-switching experiment. Switch and repeat trials differ in terms of whether participants are cued to respond to the number (repeat) or the letter (switch) on the next trial

The task-switching experiment was also identical to that used by Ophir et al. (2009). In each trial of this task, participants were presented with a fixation cross for 1,000 ms followed by a cue for 100 ms that indicated “number” or “letter.” After the cue, a number and a letter were shown adjacent to each other (see Fig. 4). When cued to respond to the number, participants had to indicate whether the number was odd (press “1” on the keyboard) or even (press the “2” key of the keyboard) as quickly as possible. When cued to respond to the letter, participants had to respond as quickly as possible to the letter by pressing “1” if the letter was a vowel and “2” if it was a consonant, with the letter being drawn from the set *A, E, I, U, P, K, N,* and *S*. The experiments consisted of four blocks of 80 trials, of which 40% were “switch” trials (number cue preceded by letter cue or vice versa) whereas the remaining trials were “repeat” trials. These two types of trials were presented in a random order.

#### Data analyses: Outcome measures and criteria for excluding observations

In this section, we describe the criteria we used for the exclusion of participants and trials, and the outcome measures we used for analyses. For all experiments, we excluded participants who performed at chance. This resulted in the exclusion of one participant from the LMM group for the change-detection task. For the other experiments, no participants were excluded on the basis of this criterion. Our exclusion criteria for trials differed across experiments, and these criteria are detailed in the sections to follow.

For the change-detection task, our analysis included only those trials in which the participant responded in time to the test array, that is, during the 2 seconds for which the test array was presented. This resulted in a loss of 4.02% of the trials. For the remaining trials we used the hit and false-alarm rates to calculate Cowan’s *K* as a measure of working memory capacity (see Cowan, 2000), with K = S * (*H-F*), where *K* is the number of targets retained in working memory, *S* is the number of elements in the memory set, and *H* and *F* are hit and false alarm rates, respectively.

For the AX-CPT, we examined the hit and false-alarm rates only for responses to the last red letter in the sequence, which would be a target in case it was an *X* that was preceded by a red *A* (AX trials) or a nontarget in all other cases (BX trials). Since Ophir et al. (2009) only found differences in response times, our analysis of these trial types also focused on response times. For these analyses, we only included those trials in which the participant’s response to first and last red letters were correct and we also excluded trials in which the response time to first and last red letters in the sequence were lower than 200 ms. This resulted in the exclusion of 40.6% of the trials,^2^ thus leaving an average of 89 trials per participant to include in our analysis.

For the *N*-back task, we ignored response times and hit rates, and instead focused the false-alarm rates because the main finding of interest in Ophir et al.’s (2009) study was an interaction effect of load (two-back vs. three-back) and group (LMM vs. HMM) on false-alarm rates, with HMMs showing a stronger in increase in false alarms with increasing load.

For the analysis of the task-switching experiment, we examined the response times for switch and repeat trials, using only those trials in which the response was correct. In addition, we examined the switch cost, which is the difference in response times for switch and repeat trials. Prior to data analysis, we removed trials with response times below 200 ms and we used van Selst and Jolicoeur’s (1994) procedure to detect outliers on the upper end of the distribution. This resulted in the exclusion of 4.07% of the trials.

### Results

Our report of the results in the main text is restricted to the analyses of the main findings of interest, listed in Table 1. We report the results of the analyses of other outcome measures and conditions in a supplementary document. In the following, we describe, per experiment, our achieved replication power for the effects of interest, followed in turn by a report of the results of applying NHST for these effects, along with the outcomes for any auxiliary effects that were tested in the same analysis (e.g., the main effects of group and distractor set size in the change-detection task, for which the prediction was a significant interaction without significant main effects; see Table 1). In addition, we report the effect sizes and their confidence intervals for all effects, and we report the outcomes of a Bayesian analysis for the seven effects of interest.

#### Change detection: Achieved replication power

For the change-detection task, we had to remove one participant from the LMM group due to chance-level performance. To calculate the achieved power we had for replicating Ophir et al.’s (2009) finding of a significant interaction group (LMM vs. HMM) and distractor set size (0, 2, 4, or 6), for the condition with a memory set size of two items, the final sample size thus consisted of 10 HMMs and 12 LMMs. Since the sample sizes differed per group, we were unable to calculate the exact power we had for our statistical test of the interaction effect, because this would require more detailed insights about the original effects than we could gain from the statistics reported for these effects. To circumvent this matter, we decided to compute a conservative power estimate, by using twice the smallest sample size for our calculations. Thus, our calculation of achieved power was based on a sample size of 2 × 10 = 20 for the change-detection task. To calculate our achieved replication power, we used G*Power 3.1. software (Faul, Erdfelder, Lang, & Buchner, 2007), and selected and set the following parameters: *F* tests, ANOVA repeated measures, within–between interaction, post hoc, effect size *f* = .344, *α* = .05, number of groups = 2, number of measurements = 4, correlation among repeated measures = .5, and nonsphericity correction *ε* = 1. This calculation showed that a conservative estimate of our replication power for the interaction effect was equal to .95.

#### Change detection: Results

**Fig. 5:**
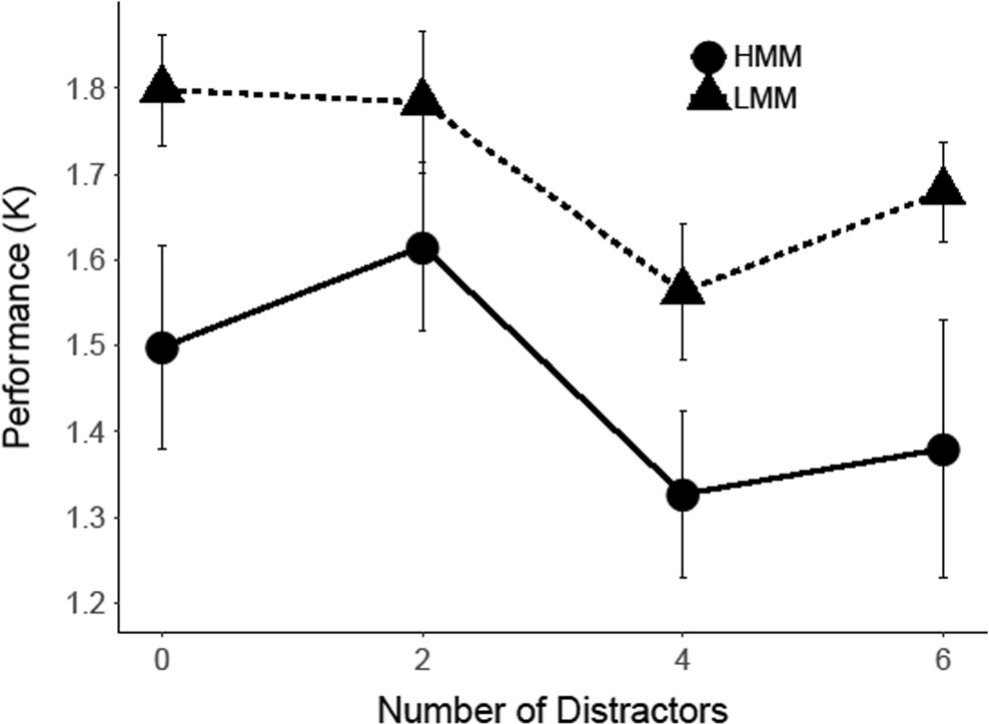
Change-detection performance for the condition with two targets and zero, two, four, or six distractors in Experiment 1. *Error bars r*epresent within-subjects standard errors of the means (Morey, 2008)

To determine whether our results replicated Ophir et al.’s (2009) finding of a Group × Distractor Set Size interaction, we conducted a repeated-measures ANOVA, with group (LMM vs. HMM) as a between-subjects factor and distractor set size (0, 2, 4, or 6) as a within-subjects factor. The analysis yielded a main effect of group, *F*(1, 20) = 6.48, *p* = .019, η_p_^2^ = .12, *d =* .74, and a main effect of distractor set size, *F*(3, 60) = 2.97, *p* = .039, η_p_^2^ = .079, *d =* .58. As can be seen in Fig. 5, the main effect of group reflected the fact that performance was worse overall for HMMs than for LMMs, and the main effect of distractor set size entailed that all participants showed a decrease in performance with increasing numbers of distractors. Most importantly, however, the results did not show a significant Group × Distractor Set Size interaction, *F*(3, 60) = 0.22, *p* = .880, η_p_^2^ = .01, and our calculation of an effect size for this interaction effect yielded a negative effect because the rate at which performance decreased across increasing distractor set sizes was higher for LMMs than HMMs, *d = −*.21, CI [−1.11, 0.69], thus demonstrating a trend in opposite direction to Ophir et al.’s (2009) finding of increased susceptibility to distraction in HMMs. A Bayes factors analysis for this interaction effect yielded a *BF*_*01*_ = 6.83, thus indicating that our experiment yielded moderate evidence for the absence of this interaction effect.

#### AX-CPT: Achieved replication power

For the AX-CPT, our primary targets for replication were the reaction times on AX and BX trials (see Table 1), for which Ophir et al. (2009) found that HMMs responded more slowly than LMMs. Replication power was calculated by entering our sample size into the G*Power 3.1. software (Faul et al., 2007), with these settings: *t* tests, difference between two independent means, post hoc, one-tail, effect size *d* = 1.19 for AX RT and 1.19 for BX RT, *α* = .05, *N*_group1_ = 10, *N*_group2_ = 13. This analysis showed that our sample size yielded a power of .86 for replicating both of these effects.

#### AX-CPT: Results

**Fig. 6:**
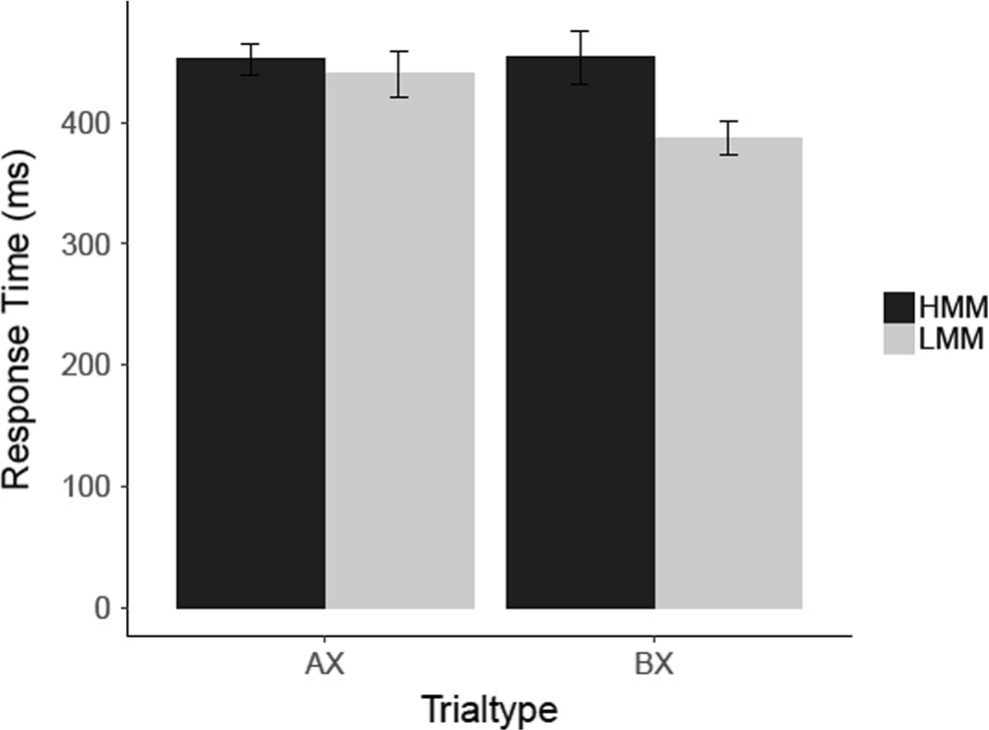
Results for the AX-CPT with distractors in Experiment 1. Mean response times (ms) are shown for correct responses to targets (AX) and nontargets (AY, BX, and BY). ***Error bars*** represent within-group standard errors of the means (Morey, 2008)

To determine if HMMs responded slower to AX and BX trials, we conducted two independent-samples *t* tests. These analyses showed that HMMs responded slower than LMMs in BX trials, *t*(21) = 1.88, *p* = .037 (one-tailed), *d* = .79, CI [−0.12, 1.70], *BF*_*10*_ = 2.42, but not on AX trials, *t*(21) = .76, *p* = .229 (one-tailed), *d* = .32 CI [−0.56, 1.20], *BF*_*01*_ = 1.43 (see Fig. 6). Thus, while the significance tests yielded evidence for a statistically significant difference in response times on BX trials only, the Bayes factors analysis showed that this effect was based on only anecdotal evidence. Likewise, the Bayes factors analysis for the nonsignificant difference in RTs on AX trials also showed that there was only anecdotal evidence in favor of the absence of this difference.

#### *N*-back: Achieved replication power

For the *N*-back task, the primary finding of interest in the study by Ophir et al. (2009) was that HMMs showed a significant increase in false alarms as memory load increased across the two-back and three-back conditions. Given that our sample sizes for the LMM and HMM groups differed (*N* = 10 and *N* = 13 for HMMs and LMMs, respectively), we decided to calculate a conservative power estimate using a sample size of 10 participants per group. The analysis in G*Power 3.1. (Faul et al., 2007) was done with these settings: *F* tests, ANOVA repeated measures, within–between interaction, post hoc, effect size *f* = .423, *α* = .05, number of groups = 2, number of measurements = 2, correlation among repeated measures = .5, and nonsphericity correction *ε* = 1. This conservative estimate of our replication power had a value of .95, thus signifying a more than acceptable level of power for this test (e.g., Cohen, 1992).

#### *N*-back task: Results

**Fig. 7:**
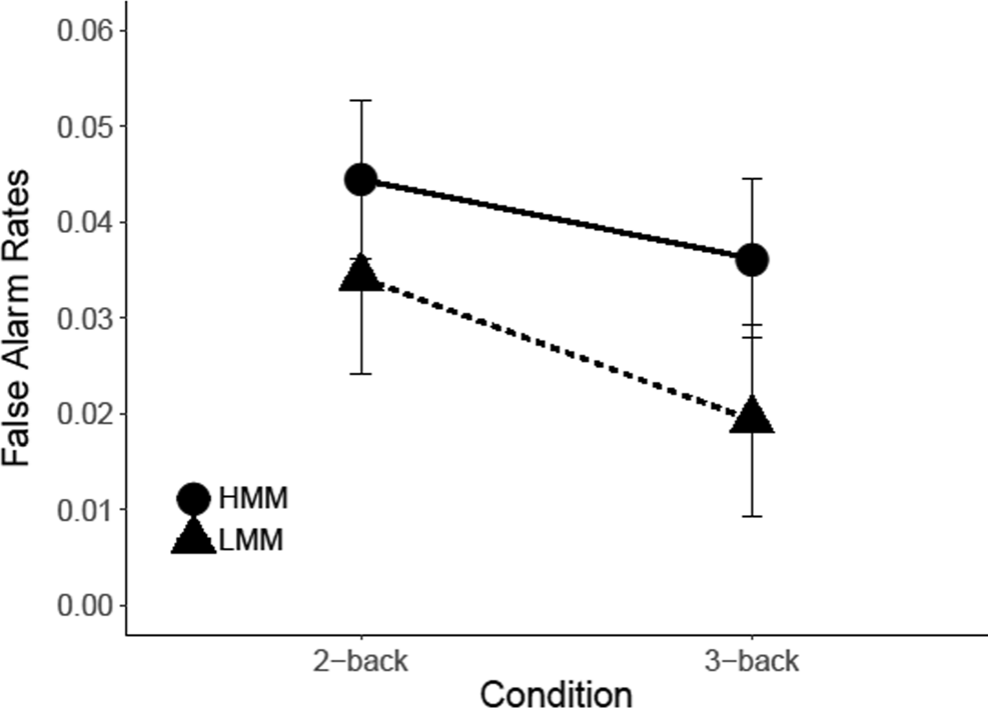
Results *N*-back task. False alarm rates are plotted as a function of WM load (two-back vs. three-back) and Group (LMM vs. HMM). *Error bars* represent within-group standard errors of the means (Morey, 2008)

Figure 7 shows the false-alarm rates of LMMs and HMMs for the two-back and three-back conditions. In analyzing these results, we conducted a repeated-measures analysis of variance, with group (LMM vs. HMM) as a between-subjects factor and WM load (two-back vs. three-back) as a within-subjects factor. The results showed no significant main effect of WM load, *F*(1, 21) =.97, *p* = .335, η_p_^2^ = .044, and no main effect of group, *F*(1, 21) = .96, *p* = .338, η_p_^2^ =.044. More importantly, the critical Group × WM Load interaction also failed to reach significance, *F*(1, 21) = .08, *p* = .781, η_p_^2^ < .001, *d =* .13, CI [−0.75, 1.01], *BF*_*01*_ = 2.6.

#### Task switching: Achieved replication power

For the task-switching experiment, Ophir et al. (2009) found that HMMs were significantly slower to respond on both switch and repeat trials, and that they also showed a significantly larger switch cost, defined in terms of the difference in RT between switch and repeat trials. Replication power for these three effects was computed in G*Power (Faul et al., 2007), with the following settings: settings: *t* tests; difference between two independent means; post hoc; one-tail; effect size *d* = .97 for switch RT, .83 for repeat RT, and .96 for switch cost; *α* = .05; *N*_group1_ = 10; *N*_group2_ = 13. These analyses showed that our sample size of 10 HMMs and 13 LMMs yielded a power of .72, .60, and .71, respectively, for replicating Ophir et al.’s finding of a difference in switch RT, repeat RT, and switch cost.

#### Task switching: Results

**Fig. 8:**
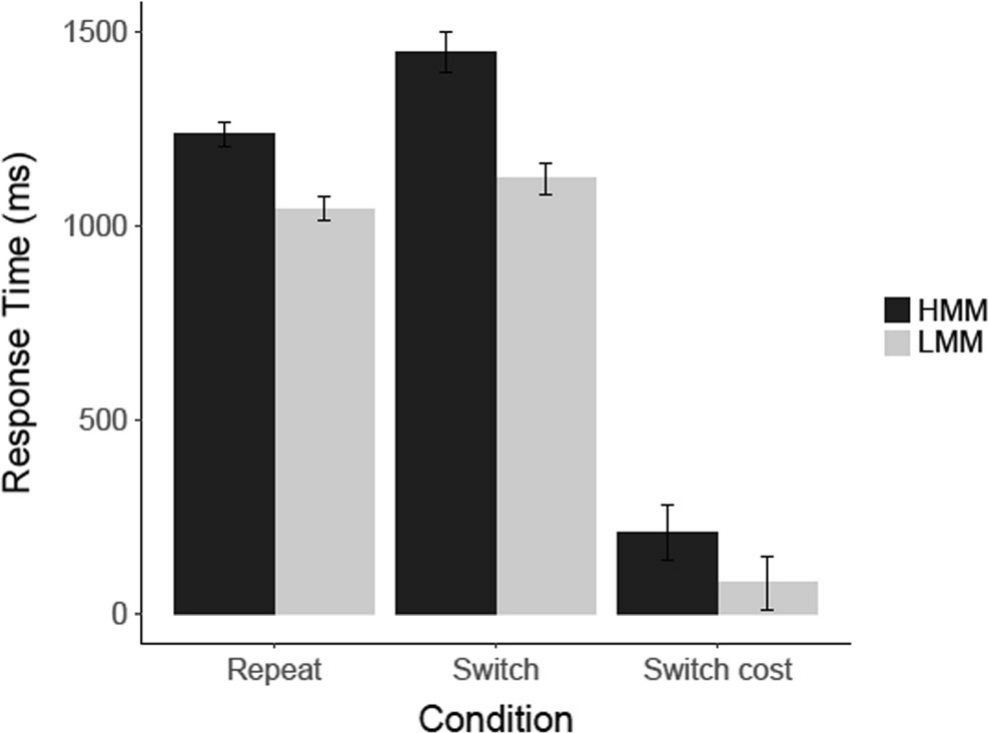
Results for the task-switching experiment in Experiment 1. Mean response time (ms) is shown for correct responses on switch and repeat trials, for HMMs and LMMs separately. *Error bars* represent within-group standard errors of the means

The results of our task-switching experiment are shown in Fig. 8. An analysis of these results showed that, compared to LMMs, HMMs were slower in switch trials, *t*(21) = 2.0, *p* = .029 (one-tailed), *d* = .84, CI [−0.07, 1.75], *BF*_*10*_ = 2.84, and they had a larger switch cost, *t*(12.33, corrected for inequality of variance) = 2.97, *p* = .006 (one-tailed), *d* = 1.35, CI [0.38, 2.32], *BF*_*10*_ = 20.1. However, we did not find that HMMs were also slower in the repeat trials, *t*(21) = 1.43, *p* = .083 (one-tailed), *d* = .60, CI [−0.29, 1.49], *BF*_*01*_ = .72.

### Discussion

In Experiment 1, we tested the replicability of the seven findings that we identified as being the key findings that led Ophir et al. (2009) to conclude that heavy media multitasking is associated with increased susceptibility to distraction. In testing the replicability of these findings, we copied the methods used by Ophir et al., we used a sample size that yielded an adequate level of power (Cohen, 1992), and we used the a rigorous approach to statistical analysis, such that we used a combination of power analyses, NHST, effect sizes, and Bayes factors in examining the outcomes of our replication study. By implication, we can assess the success versus failure of our replication studies in terms of different metrics (see also, Open Science Collaboration, 2015).

To start, one can evaluate the results of our first replication study in terms of the achieved replication power—that is, the likelihood that we would replicate the effects of Ophir et al., given our sample sizes, and assuming that the effects found by Ophir et al. were true—and statistical significance. From this perspective, a first point of consideration is that the results of our power analyses showed that our tests had an average replication power of .81, which is generally considered an acceptable level of power (Cohen, 1992), and which means that one would expect that if the seven effects reported by Ophir et al. were true, then at least five of these seven effects (i.e., 81% of the seven effects tested) would be replicated at α = .05 in the current replication study. This turned out not to be the case, as only three of the seven effects reached significance in our replication study. Specifically, HMMs were significantly slower than LMMs in responding to BX probes in the AX-CPT, they were significantly slower than LMMs in responding on switch trials in the task-switching experiment, and they showed a larger switch cost than LMMs in the task-switching experiment. On the other hand, we did not find a significant difference in response times on AX trials in the AX-CPT, we did not find a difference in false alarms in the *N*-back task, we did not find a difference in vulnerability to distraction in the change-detection task, and we also did not find a difference in response times on repeat trials in the task-switching experiment.

When evaluating the results of our replication study on the basis of Bayes factors, we find that only one of the three statistically significant effects—the finding of a greater switch cost in HMMs—was based on strong evidence, whereas the effects for response times on BX trials in the AX-CPT, and for switch trials in the task-switching experiment were based on only anecdotal evidence. Importantly, however, the Bayes factors also showed that only one of the four nonsignificant effects yielded moderate evidence in favor of the null hypothesis, and this concerned the absence of an interaction effect of media multitasking and distractor set size in the change detection task. Thus, according to the Bayesian analyses, our replication attempt was largely indecisive, as only two of the seven effects of interest produced clear evidence for the presence or absence of an effect.

**Fig. 9:**
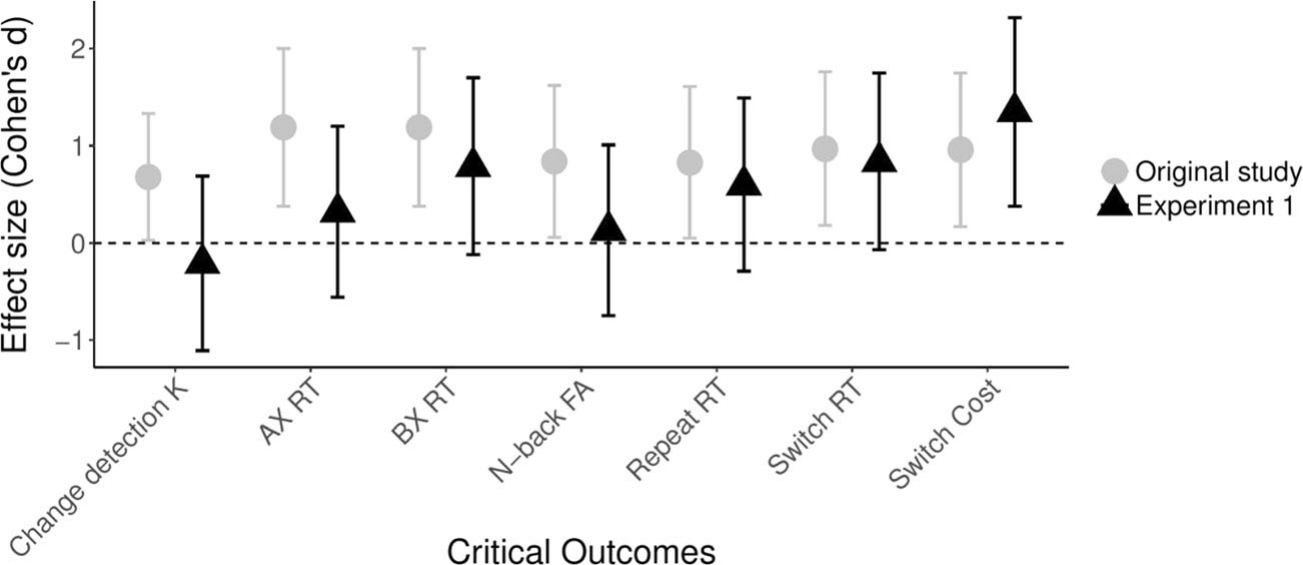
Comparison of effect sizes (Cohen’s *d*) and their 95% confidence intervals for the seven effects of interest in Ophir et al. (original study) and in our first replication study (Experiment 1)

Moving beyond the binary diagnosis of the presence versus absence of effects in terms of statistical significance or *BF* > 3, we can also evaluate the outcomes of our replication study by considering the corresponding effect sizes and their confidence intervals. This evaluation moves beyond the diagnosis of presence versus absence of effects, as it sheds light on the strength of these effects. When comparing the effect sizes we obtained in our seven replication tests to those found by Ophir et al. (see Fig. 9), we find that the average effect size for the replication tests was markedly lower than the average size of these effects in Ophir et al. (*M* = 0.55, *SD* = .51 vs. *M* = 0.95, *SD* = .19, respectively). At the same time, however, all of the effects found by Ophir et al. fell within the 95% confidence interval of the replication effect sizes, and, except for the outcome of the change-detection task, all other replication tests yielded evidence for an effect in the same direction as the effects found by Ophir et al. Thus, when considering effect size, the results of our first replication study can be said to conform largely to the outcomes of Ophir et al., with the qualification that the effects were smaller in the current replication study.

## Experiment 2

Taken together, we can conclude that the results of our first replication study did not produce a successful replication in terms of statistical tests aimed at determining the presence of an effect (i.e., power analysis, NHST, and Bayes Factors), as these metrics showed that we replicated fewer effects than would be expected if the effects of Ophir et al. were true. At the same time, however, six out of seven replication tests did show an effect in the same direction as the effects found by Ophir et al. (2009), but these effects were markedly smaller than those observed by Ophir et al. In considering the possible reasons for why our first replication study generally produced smaller effects than those found by Ophir et al. (2009), an interesting possibility can be found in the fact that the Indonesian participants in our first replication study generally scored much higher on the Media Multitasking Index (MMI) than the participants in most previous studies that used the MMI, including the study by Ophir et al. Specifically, the average MMI for participants in Ophir et al.’s studies was 4.38, whereas it was 6.80 in our study. Accordingly, one could argue that perhaps our finding of smaller effects might have been because our participants in the first replication study had unusually high MMI scores. Since previous work suggests that, compared to participants from Western countries such as Britain and the U.S., Indonesian participants have the tendency to use more extreme answer alternatives in completing surveys (Stening & Everett, 1984), we addressed this possibility by running a second replication study using participants from the University of Groningen, The Netherlands. Aside from providing a second attempt at replication of Ophir et al.’s findings, our second replication study also aimed to shed light on the reliability of the MMI, by including a second administration of the media-use questionnaire so as to enable an assessment of the test–retest reliability of this questionnaire.

### Methods

#### Participants

A total of 306 students from the University of Groningen, The Netherlands, were asked to complete the Media Multitasking Index questionnaire, and 205 of these participants indeed completed the questionnaire. The MMI scores for these 205 participants were normally distributed, Kolmogorov–Smirnov, *Z* = .99, *p* = .28, with a mean of 3.80 and a standard deviation of 1.89. This distribution of scores was comparable to that in the study by Ophir et al. (2009), which had a mean 4.38 and a standard deviation of 1.52. Of our 205 participants, 52 were classified as HMM and 52 were classified as LMM, based on the fact that their scores fell within the lower and upper quartiles of the distribution of scores. Of these 104 participants, 19 HMMs (*M* = 6.63, *SD* = 1.40) and 11 LMMs (*M* = 1.61, SD = .64) responded to our invitation to take part in a lab study in return for monetary compensation or course credits.

#### Materials, procedures, and data analysis

The second replication study was identical to the first replication in all regards, except for the fact that the experiments for the second study were run in isolated experimental booths, using a program written in E-Prime Version 2.0 (MacWhinney, St James, Schunn, Li, & Schneider, 2001), with the stimuli being presented on a 17-inch CRT monitor that was controlled by an Intel i3, 3.4 GHz CPU with 8 GB of RAM. In addition, the second replication study differed from the first in that participants were asked to fill in the media-use questionnaire for a second time at the start of the lab study, thus enabling us to compute the test–retest reliability of the questionnaire. The second administration of the questionnaire in the lab study took place approximately 1 week after participants had first filled it in. The exclusion of participants and trials was done according to the same rules as those used in the first study, and the exclusion of participants and trials is described in detail per experiment in the following sections.

### Results

#### Test–retest reliability of the MMI

To determine the reliability of the MMI, we computed the test–retest correlation for the participants who took part in the lab study. This analysis showed that the correlation between the repeated administrations of the questionnaire was high, with *r*(28) = .93, *p <* .01.

#### Change-detection task: Achieved replication power

For the change-detection task, we had to remove one participant from the HMM group due to chance-level performance, thus yielding a final sample size of 18 HMMs and 11 LMMs. To calculate our power for replicating Ophir et al.’s (2009) finding of an interaction between media multitasking and distractor set size, we entered a sample size of 2 × 11 = 22 into G*Power 3.1. (Faul et al., 2007), with the following settings: *F* tests, ANOVA repeated measures, within–between interaction, post hoc, effect size *f* = .344, *α* = .05, number of groups = 2, number of measurements = 4, correlation among repeated measures = .5, and nonsphericity correction *ε* = 1. This calculation showed that our sample size for the change-detection task yielded a replication power of .97 for finding the Group × Distractor Set Size interaction effect reported by Ophir et al.

#### Change detection task: Results for two-target condition

**Fig. 10:**
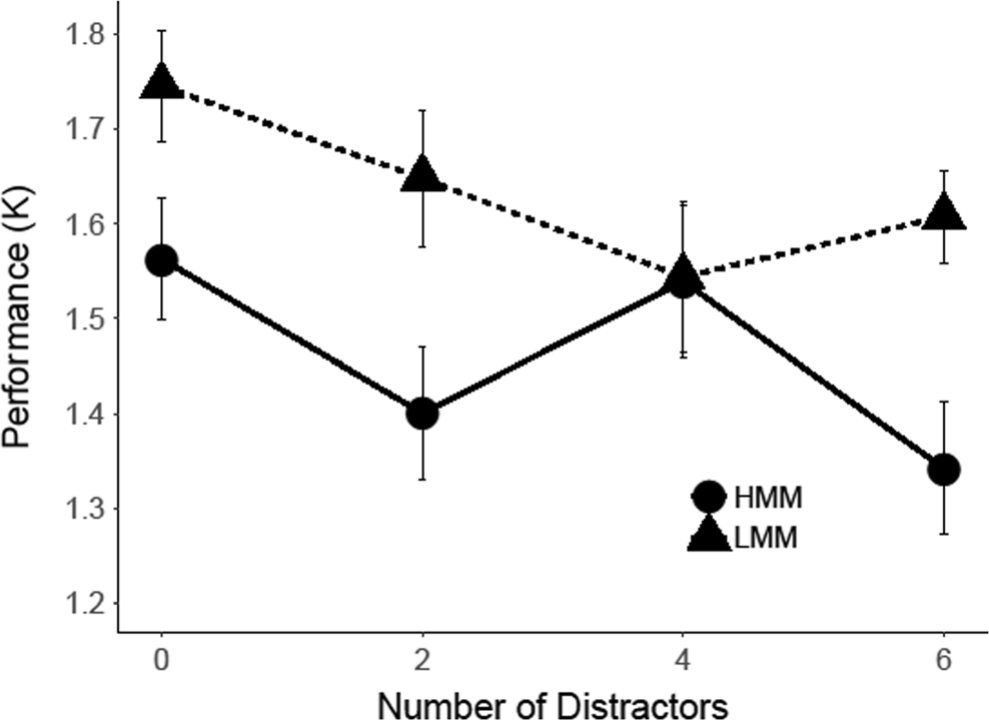
Change detection performance for the condition with two targets and zero, two, four, or six distractors in Experiment 2. *Error bars* represent within-subjects standard errors of the means (Morey, 2008)

For the condition with a memory set of two items, we examined Cowan’s *K* as a function of group and distractor set size (0, 2, 4, or 6; see Fig. 10). The analysis showed no significant main effect of group, *F*(1, 27) = 3.29, *p* = .081, η_p_^2^ = .06, *d =* .51, or of distractor set size, *F*(3, 81) = 2.08, *p* = .110, η_p_^2^ = .03, *d =* .35. In addition, the results did not show an interaction between group and distractor set size, *F*(3, 84) = 1.29, *p* = .284, η_p_^2^ = .02, *d =* .43, CI [−0.36, 1.22], *BF*_*01*_ = 2.69.

#### AX-CPT with distractors: Achieved replication power

For the AX-CPT, we had to remove 10 participants due to poor performance. These participants appeared to have failed to understand the task instructions, as they had an accuracy of zero in one of the conditions. Exclusion of these participants entailed that the subsequently reported analyses of performance in the AX-CPT were conducted with a sample of 14 HMMs (*M*_MMI_ = 6.48, *SD* = 1.29) and six LMMs (*M*_MMI_ = 1.5, *SD* = 0.76). To calculate our achieved replication power for replicating Ophir et al.’s (2009) finding that HMMs showed increased RTs on AX and BX trials, this sample size was entered into the G*Power 3.1 (Faul et al., 2007) with these settings: *t* tests, difference between two independent means, post hoc, one-tail, effect size *d* = 1.19 for AX RT and 1.19 for BX RT, *α* = .05, *N*_group1_ = 14, *N*_group2_ = 6. These calculations showed even with this small sample of participants, we still had a power of .76 for replicating the results Ophir et al. found in their analyses of RT for AX and BX trials.

#### AX-CPT with distractors: Results

**Fig. 11:**
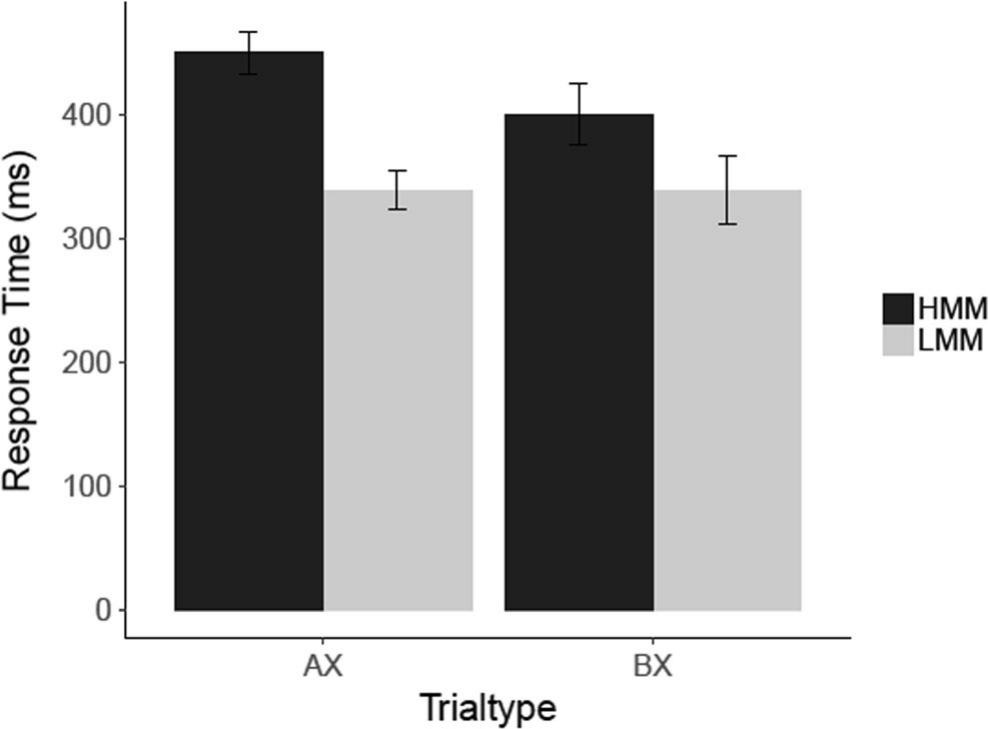
Results for the AX-CPT with distractors in Experiment 2. Mean response times (ms) are shown for correct responses to AX and BX trials. *Error bars* represent within-group standard errors of the means (Morey, 2008)

To compare the response times of HMMs and LMMS to AX and BX trials in the AX-CPT, we conducted two independent-samples *t* tests (see Fig. 11 for the results). These analyses showed that HMMs were slower in AX trials, *t*(18) = 2.58, *p* = .009 (one-tailed), *d* = 1.26, CI [0.15, 2.37], *BF*_*10*_ = 6.36, but not in BX trials, *t*(18) = .98, *p* = .169 (one-tailed), *d* = .48, CI [−0.56, 1.52], *BF*_*01*_ = 1.09.

#### *N*-back task: Achieved replication power

For the *N*-back task, we had to remove two participants from the HMM group and two participants from the LMM group due to poor performance, thus resulting in a final sample size of 17 HMMs and nine LMMs. The reasons for excluding these participants were that one participant did not respond to any of the trials, two participants did not respond to more than half of the trials, and one participant had a higher false alarm than hit rate. To calculate our power for replicating Ophir et al.’s (2009) finding of an interaction between load (two-back vs. three-back) and group (HMM vs. LMM) on false-alarm rates, we set the sample size to 2 × 9 = 18 for obtaining a conservative power estimate. Power calculation was done in G*Power 3.1., with these settings: *F* tests, ANOVA repeated measures, within–between interaction, post hoc, effect size *f* = .423, *α* = .05, number of groups = 2, number of measurements = 2, correlation among repeated measures = .5, and nonsphericity correction ε = 1. This calculation showed that our sample of participants entailed that we had a replication power of .92 for replicating Ophir et al.’s finding of an interaction of group and memory load on false-alarm rates.

#### *N*-back task: Results

**Fig. 12:**
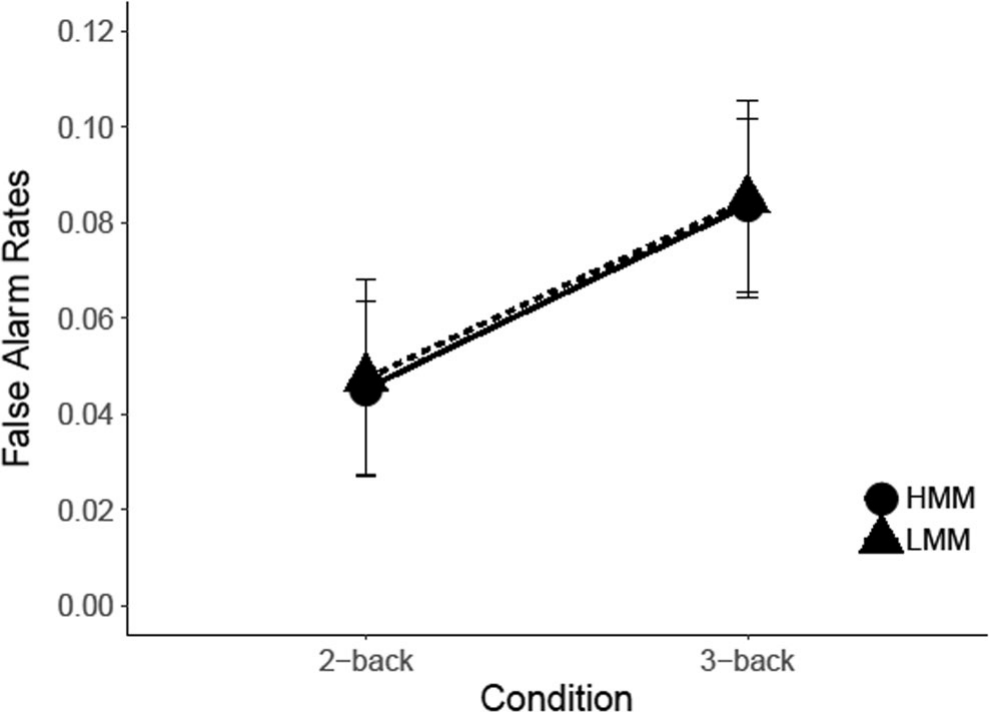
Results *N*-back. False-alarm rates are plotted as a function of WM load (two-back vs. three-back) and group (LMM vs. HMM). *Error bars* represent within-group standard errors of the means (Morey, 2008)

An analysis of the false-alarm rates (see Fig. 12) as a function of group (HMM vs. LMM) and memory load (two-back vs. three-back) showed no significant main effect of WM Load, *F*(1, 24) = 3.38, *p* = .078, η_p_^2^ = .123, and no main effect of group, *F*(1, 24) *=* .003, *p =* .954, η_p_^2^ < .001. In addition, the interaction of Group × WM Load failed to reach significance, *F*(1, 24) < .001, *p* = .982, η_p_^2^ < .01, *d <.*01, CI [−0.85, 0.85], *BF*_*01*_ = 2.46.

#### Task switching: Achieved replication power

To calculate our power for replicating Ophir et al.’s (2009) findings that HMMs showed larger switch costs and higher RTs on repeat and switch trials for the task-switching experiment, we entered our sample size of 19 HMMs and 11 LMMs into G*Power 3.1. (Faul et al., 2007), using these settings: *t* tests; difference between two independent means; post hoc; one-tail; effect size *d* = .97 for switch RT, .83 for repeat RT, and .96 for switch cost; *α* = .05; *N*_group1_ = 19; *N*_group2_ = 11. These calculations showed that our sample yielded replication powers of .80, .69, and .79, for the effects Ophir et al. found for switch RT, repeat RT, and switch cost, respectively.

#### Task switching: Results

**Fig. 13:**
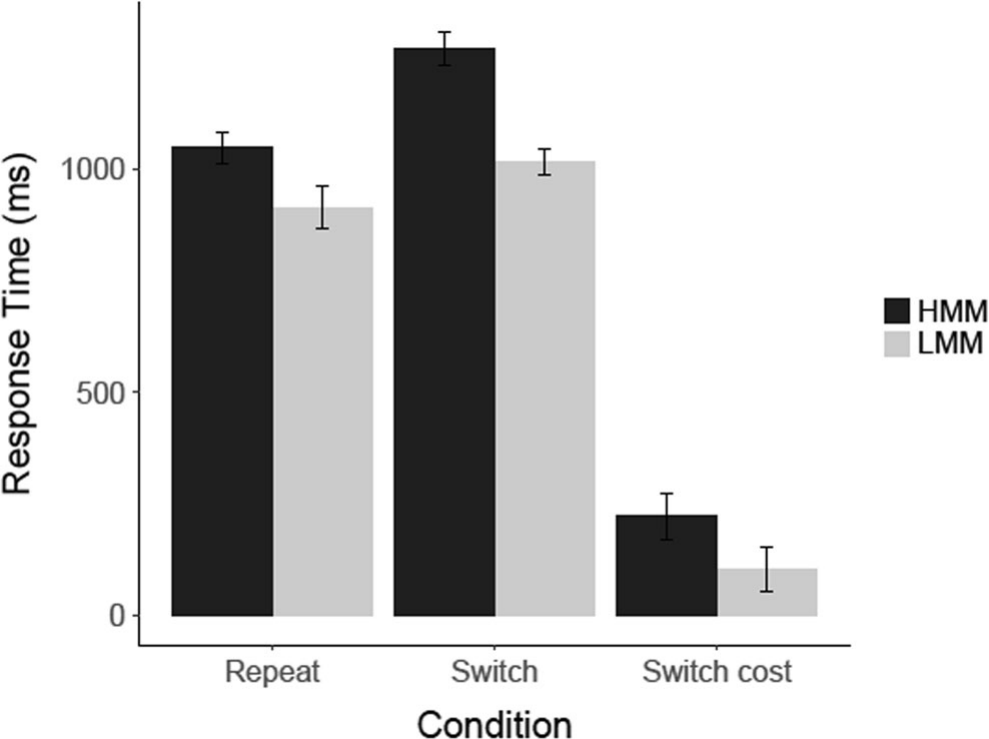
Results for the task-switching experiment in Experiment 2. Mean response time (ms) is shown for correct responses on switch and repeat trials, for HMMs and LMMs separately. *Error bars* represent within-group standard errors of the means

The results for the task-switching experiment are shown in Fig. 13. The analyses showed that HMMs were significantly slower than LMMs in switch trials, *t*(28) = 1.73, *p* = .047 (one-tailed), *d* = .66. CI [−0.14, 1.46], *BF*_*10*_ = 1.93. The analyses of switch costs and response times on repeat trials showed no statistically significant difference, with *t*(28) = 1.21, *p* = .117 (one-tailed), *d* = .46, CI [−0.33, 1.25], *BF*_*01*_ = 0.95, and *t*(28) = 1.66, *p* = .054 (one-tailed), *d* = .63, CI [−0.16, 142], *BF*_*01*_ = 1.79.

### Discussion

**Fig. 14:**
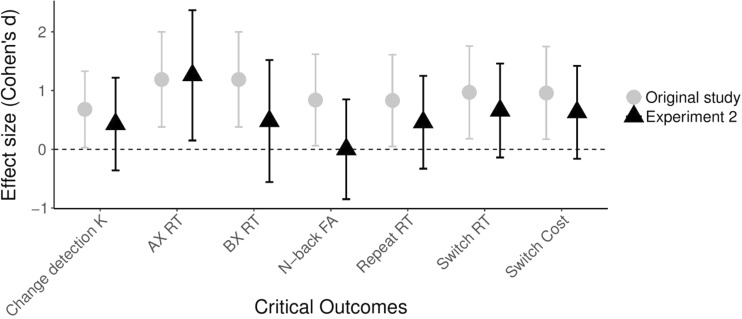
Overview of the results of our second replication study. Effect sizes (Cohen’s *d*) and their 95% confidence intervals are shown for the seven effects of interest in Ophir et al. (original study) and in our second replication study (Experiment 2)

Aside from demonstrating that the MMI has a high test–retest reliability (see also, Baumgartner, Lemmens, Weeda, & Huizinga, 2016), the results from our second replication study largely conform to those obtained in our first replication study. Specifically, our tests of the replicability of Ophir et al.’s (2009) main findings had an average replication power of .81, yet only two out of seven findings yielded a statistically significant outcome in the same direction as that found by Ophir et al. Specifically, HMMs were slower in AX trials of the AX-CPT task and they were slower than LMMs on switch trials. In terms of Bayes factors, our analyses showed that the difference in AX trials was based on moderately strong evidence, whereas the difference on switch trials was based on only anecdotal evidence. In addition, the BFs showed that all of the nonsignificant effects involved only anecdotal evidence in favor of the null hypothesis. As for the effect sizes (see Fig. 14), the results of our second replication study showed that all effects were in the same direction as those found by Ophir et al., with HMMs performing worse than LMMs. However, as in our first replication study, the effects in the second replication study were again smaller than those found by Ophir et al. (with *M* = 0.56, *SD* = .37 vs. *M* = 0.95, *SD* = .19, respectively). Accordingly, it can be concluded that the results of our second replication generally conform to those of our first replication study in suggesting that while HMMs may indeed perform worse than LMMs on various tests of distractibility, the magnitude of these differences is smaller than the effects found by Ophir et al.

## Meta-analysis

Taken together, the results of our replication studies can be said to provide only partial support for the existence of an MMI–distractibility link, as the majority of our significance tests and Bayes factors analyses did not yield convincing support for the existence of this link, but the outcomes did generally show effects in the same direction as those found by Ophir et al. (2009). As a final step in our examination of the MMI–distractibility link, we aimed to arrive at a proper estimate of the strength of the relationship between media multitasking and distractibility in laboratory tests of information processing. To this end, we conducted a meta-analysis that included the results of the current replication studies along with those of all previous studies that have used similar laboratory tasks to investigate the relationship between media multitasking and distractibility, including the seminal study by Ophir et al. (2009). By calculating a weighted mean effect size on the basis of the results of all studies done to date, this analysis can provide the most sensitive and powerful test of the existence and strength of the MMI–distractibility link. In addition, we also made use of moderator analyses to determine whether the MMI–distractibility link differed across certain subsets of tasks or participants, and we used meta-analytical tools to diagnose and correct for the presence of any small-study effects (i.e., the influence of the presence of relatively many small studies that showed large, positive effects, and relatively few, similarly small studies with negative or null effects; Duval & Tweedie, 2000; Egger, Davey Smith, Schneider, & Minder, 1997; Peters, Sutton, Jones, Abrams, & Rushton, 2007; Sterne et al., 2011; Thompson & Sharp, 1999).

### Methods

#### Criteria for study inclusion

We aimed to include all published studies that examined the relationship between media multitasking and distractibility in laboratory tasks such as those used in the original study by Ophir et al. (2009). Accordingly, our inclusion criteria for the meta-analysis were that the study in question should include a statistical test of this relationship, either in the form of a between-groups comparison of LMMs and HMMs, or in the form of a correlation between media multitasking and performance on one or more laboratory tests of distractibility in information processing. In determining which tasks can be considered to provide an index of distractibility, we adopted a categorization and definition of distractibility similar to that used by Ophir et al. in their interpretation of their findings. Specifically, we selected tasks in which participants were asked to respond to target stimuli that were presented under conditions in which distraction could either be caused by irrelevant stimuli that were presented simultaneously or before or after the target in a particular trial (environmental distraction), or by irrelevant stimuli held in memory (memory-based distraction), or by an irrelevant, previously used task set (task-set distraction). Accordingly, any task that involved the sequential or simultaneous presentation of one or more targets and one or more distractors would be considered an index for vulnerability to environmental distraction, whereas any task that involved the possibility of distraction from previously memorized stimuli would be considered an index of vulnerability to memory-based distraction, and any task that involved a comparison of performance with or without a task-switch would be considered as an index of distraction caused by a previously used task set.

#### Literature search and studies included

The search for studies on the relationship between media multitasking and distractibility was done using the PsycInfo, ERIC, Medline, and CMMC databases, with a combination of the following keywords: media multitasking* AND (cognitive control* OR working memory* OR attention*). This search yielded a total of 40 published articles, of which 12 included one or more experiments that met our selection criteria (Alzahabi & Becker, 2013; Baumgartner et al., 2014; Cain et al., 2016; Cain & Mitroff, 2011; Cardoso-Leite et al., 2015; Gorman & Green, 2016; Minear et al., 2013; Moisala et al., 2016; Ophir et al., 2009; Ralph & Smilek, 2016; Ralph, Thomson, Seli, Carriere, & Smilek, 2015; Uncapher et al., 2015). Aside from these published studies, we also included the effect sizes from Experiments 1 and 2 of the current study. These studies are listed in Table 2, along with the type of task that was used in the study, the type of distraction that was involved in this task, and the distractibility effect that was used for computing the effect size.

**Table 2:** Studies and effects included in the meta-analysis

Distraction type	Study (year, experiment)	*N*_HMM_	*N*_LMM_	*N*_tot_	Task	Outcome ~ predictor
Environmental	Cardoso-Leite et al. (2015)	12	20	32	Change detection	*K* ~ Ndist * MMI
	Gorman & Green (2016)	22	20	42	Change detection	*d’* ~ Ndist * MMI
	Ophir et al. (2009, Exp. 1)	19	22	42	Change detection	*K* ~ Ndist * MMI
	Uncapher et al. (2015)	36	36	72	Change detection	*K* ~ Ndist * MMI
	Uncapher et al. (2015)	36	36	72	Change detection	*K* ~ Ndist * MMI
	Wiradhany and Nieuwenstein (2016, Exp. 1)	10	12	22	Change detection	*K* ~ Ndist * MMI
	Wiradhany and Nieuwenstein (2016, Exp. 2)	18	11	29	Change detection	*K* ~ Ndist * MMI
	Cardoso-Leite et al. (2015)	12	20	32	AX-CPT	Avg. RT ~ MMI
	Ophir et al. (2009, Exp. 3)	15	15	30	AX-CPT	AX-RT ~ MMI
	Wiradhany and Nieuwenstein (2016, Exp. 1)	10	13	23	AX-CPT	AX-RT ~ MMI
	Wiradhany and Nieuwenstein (2016, Exp. 2)	14	6	20	AX-CPT	AX-RT ~ MMI
	Baumgartner et al. (2014)	–	–	523	Eriksen flanker	Flanker congruency ~ MMI
	Gorman and Green (2016)	22	20	42	Eriksen flanker	Flanker congruency ~ MMI
	Minear et al. (2013, Exp. 3)	27	26	53	Eriksen flanker	Flanker congruency ~ MMI
	Ralph et al. (2015, Exp. 1)			76	SART	RT ~ MMI
	Ralph et al. (2015, Exp. 2)			143	SART	RT ~ MMI
	Ralph et al. (2015, Exp. 3)			109	Inverted SART	RT ~ MMI
	Cain & Mitroff (2011)	17	17	34	Visual search	RT ~ MMI
	Cain et al. (2016)			69	WM filtering: Count span	Accuracy ~ MMI
	Cain et al. (2016)			58	WM filtering: Recall	Accuracy ~ Ndist * MMI
	Gorman and Green (2016)	22	20	42	Test of variables of attention	RT ~ MMI
	Moisala et al. (2016)	–	–	149	Cross-modal filtering	Accuracy ~ MMI
Memory based	Cain et al. (2016)			58	*N*-back	3-back FA ~ MMI
	Cardoso-Leite et al. (2015)	12	20	32	*N*-back	3-back FA ~ MMI
	Ophir et al. (2009, Exp. 2)	15	15	30	*N*-back	FA ~ WM load * MMI
	Ralph and Smilek (2016)			265	*N*-back	3-back FA ~ MMI
	Ralph and Smilek (2016)			265	*N*-back	3-back FA ~ MMI
	Wiradhany and Nieuwenstein (2016, Exp. 1)	10	13	23	*N*-back	FA ~ WM Load * MMI
	Wiradhany and Nieuwenstein (2016, Exp. 2)	17	9	26	N-back	FA ~ WM Load *MMI
Task set	Alzahabi and Becker (2013, Exp. 1)	–	–	80	Task switching	Switch cost ~ MMI
	Alzahabi and Becker (2013, Exp. 2)	–	–	49	Task switching	Switch cost ~ MMI
	Baumgartner et al. (2014)	–	–	523	Task switching	Switch cost ~ MMI
	Cardoso-Leite et al. (2015)	12	20	32	Task switching	Switch cost ~ MMI
	Gorman and Green (2016)	22	20	42	Task switching	Switch cost ~ MMI
	Minear et al. (2013, Exp. 3)	27	26	53	Task switching	Switch cost ~ MMI
	Minear et al. (2013, Exp. 1)	33	36	69	Task switching	Switch cost ~ MMI
	Ophir et al. (2009, Exp. 3)	15	15	30	Task switching	Switch cost ~ MMI
	Wiradhany and Nieuwenstein (2016, Exp. 1)	10	13	23	Task switching	Switch cost ~ MMI
	Wiradhany and Nieuwenstein (2016, Exp. 2)	18	12	30	Task switching	Switch cost ~ MMI

*Note.* Distraction type = type of distraction involved in the study; *N*_HMM_ = sample size HMM group; *N*_LMM_ = sample size LMM group; *N*_tot_. = total sample size; Outcome = dependent variable; Predictor =effect tested in study

#### Selection of outcome variables

In selecting the outcomes for inclusion in our meta-analysis, we chose to avoid the intricacies involved in modeling multilevel dependencies that would exist due to the varying strengths of correlations between outcomes obtained from different trial types in the same task (i.e., RTs for AX and BX trials, switch costs and RTs for switch and repeat trials in a task-switching experiment) and between outcomes obtained on different tasks for the same sample of participants (e.g., distractibility in the *N*-back task and distractibility in the change-detection task). To this end, we chose to select one outcome per task, and we used a procedure for robust variance estimation to correct for variance inflation stemming from the inclusion of correlated observations for different tasks done by the same participants (Hedges, Tipton, & Johnson, 2010; Scammacca, Roberts, & Stuebing, 2014).

Specifically, for the AX-CPT, we chose to include the response times for AX trials, as this type of trial can be considered a more reliable index of performance because it occurs more frequently in the task than the BX trials.^3^ For studies on task switching, we reasoned that, compared to RTs on switch and repeat trials, the switch cost constitutes the most straightforward index of interference caused by a previously used task set, and hence we chose to only the switch cost, and not the average RTs on switch or repeat trials.

For studies using different tasks than those used by Ophir et al. (2009), we selected the outcome measure that best reflected the participant’s performance in the presence of environmental, memory-based, or task-set based distraction. Specifically, for the Sustained Attention to Response Task (SART; Ralph et al., 2015) and Test of Variables of Attention (TOVA; Gorman & Green, 2016) we used response times to targets that were shown in a sequence of distractors. Likewise, for studies using the Eriksen flanker task (Baumgartner et al., 2014; Gorman & Green, 2016; Minear et al., 2013), we chose to use the flanker congruency effect for response times to the target, which reflects the difference in RTs when targets are flanked by congruent or incongruent distractors, with larger congruency effects being indicative of greater vulnerability to distraction. For the cross-modal filtering task used by Moisala et al. (2016), we used the correlation between the MMI and accuracy in conditions in which distractors were presented in a different sensory modality than the targets. For the count-span and working-memory filtering tasks of Cain et al. (2016), we used recall performance for conditions in which the to-be-remembered targets were shown together with distractors. Lastly, for the visual-search task used by Cain and Mitroff (2011), we included the results for a test of an interaction effect of the presence vs. absence of a singleton distractor and group (HMM vs. LMM).

#### Effect-size calculation

Effect sizes were calculated in term of *Cohen’s d* (Cohen, 1988, 1992), with positive values denoting evidence for greater vulnerability to distraction in HMMs and negative values denoting an effect in opposite direction. In case of comparisons involving a within-group factor, such as the change detection task with different numbers of distractors, we first calculated partial eta squared using the equation below (Cohen, 1988; Lakens, 2013):

$$ {\eta}_P^2=\frac{F\times {df}_{effect}}{F\times {df}_{effect}+{df}_{error}}. $$

Assuming a minimum variability in the repeated measures, the partial eta squared was then transformed into a standardized mean difference using the equation (see Cohen, 1988):

$$ d=\sqrt{\frac{\eta_P^2}{1-{\eta}_P^2}\times 2k}, $$

with *k* denoting the number of between-group levels.

#### Meta-analysis: Testing the MMI–distractibility link

To determine the effect size for the association between media multitasking and distractibility, we used a random-effects model in which the overall effect size is computed from effect sizes weighted by the inverse of their variance (Borenstein, Hedges, Higgins, & Rothstein, 2009). This model was calculated in R using the metafor package (Viechtbauer, 2010). Calculation of a random-effects model increases statistical power by reducing the standard error of the weighted average effect size (Cohn & Becker, 2003). Using this method, one obtains a weighted average effect size and can assess the statistical significance of this effect.

#### Moderator analyses

Aside from examining the strength and significance of the association between media multitasking and distractibility across all studies included in the meta-analysis, we also examined whether the strength of this link was different for studies employing tasks with different types of distraction, for studies using different populations of participants, and for studies employing different statistical methods in assessing the association between media multitasking and distractibility. Specifically, we conducted three moderator analyses. In the first, we examined whether the results were different for tasks involving environmental, memory-based, or task-set distraction. In the second, we examined if the results were different depending on whether the study participants were adolescents, university students, or people from the general population. In the third, we examined if the results were different for studies in which the MMI–distractibility link was tested using either a correlational approach (i.e., resulting in a correlation coefficient that expresses the relationship between distractibility and the participants’ scores on a questionnaire measure of media multitasking), or an extreme-groups comparison based on cutoffs determined by either quartile scores or a criterion based on the standard deviation.

#### Tests and corrections for small-study effects

Lastly, we also examined whether the outcomes of the meta-analysis were influenced by small-study effects (Carter & McCullough, 2014; Duval & Tweedie, 2000; Egger et al., 1997; Peters et al., 2007; Sterne et al., 2011; Thompson & Sharp, 1999). Such effects are said to be present when the outcome of a meta-analysis is influenced by the inclusion of relatively many small-sample studies showing large, positive effects and relatively few small-sample studies showing negative or null effects. This state of affairs is typically interpreted as evidence for a reporting bias, such that researchers might refrain from attempting to publish small-sample studies showing negative or nonsignificant outcomes, and journals might likewise refrain from accepting such studies for publication. Alternatively, small-study effects can also arise due to true heterogeneity in case the small-sample studies not only differ from the larger studies in terms of sample size but also in terms of certain methodological aspects (e.g., Sterne, Gavaghan, & Egger, 2000). Accordingly, an interpretation of the presence of small-study effects requires a consideration of whether the studies employing large and small sample sizes differed in terms of certain methodological aspects, and whether the distribution of study effect sizes shows a preponderance of small-sample studies with positive, significant effects and an absence of similarly small studies showing negative or nonsignificant effects.

To evaluate the presence of small-study effects, we constructed used a contour-enhanced funnel plot in which effect sizes were plotted against a measure of their precision (i.e., standard error; Egger et al., 1997; Sterne et al., 2011; Sterne & Egger, 2001), and in which areas of statistical significance (*p* < .05) were highlighted (Peters et al., 2007; see also Carter & McCullough, 2014; Nieuwenstein, Blom, Morey, & Wicherts, 2015). In such a plot, the presence of small-study effects can be judged by determining whether the effect sizes of smaller studies with lower precision are distributed symmetrically around the estimate of the mean effect size, as would be expected when these effects are sampled from a distribution centered on the estimated mean effect size. Furthermore, by highlighting the areas of statistical significance, one can judge whether the studies that appear to be missing are studies that would have been expected to produce nonsignificant or null effects, thus allowing for an evaluation of whether the asymmetry might be due to a reporting bias (as opposed to true heterogeneity caused by differences in the design of smaller and larger studies; Peters et al., 2007). In addition to visual inspection, we also performed a regression analysis in which the standard errors of the effect sizes are used as a predictor for the effect size (Egger et al., 1997), thus offering a means to verify the presence of funnel-plot asymmetry in terms of the statistical significance of the association between effect sizes and study precision.

When small-study effects are found that are suggestive of a reporting bias, one should correct the estimated overall effect size for this bias. To this end, one can use the regression analysis to estimate the effect size of a study with maximal precision (i.e., an extrapolation to a study with a standard error of zerp; Moreno et al., 2009), or one can apply the so-called trim-and-fill procedure to fill in any effects that appear to missing in the asymmetrical funnel plot (Duval & Tweedie, 2000). While there is ongoing debate about whether these procedures lead to a proper overall estimate of effect size, there is consensus that these procedures can be used as sensitivity tests to determine the extent to which the outcome of a meta-analysis is dependent on the presence of small-study effects. Accordingly, we planned to conduct these corrective procedures in case an asymmetry suggestive of reporting bias was present, thus allowing for a further evaluation of the existence and strength of the association between media multitasking and distractibility.

### Results

#### Forest plot and results random-effect model

**Fig. 15:**
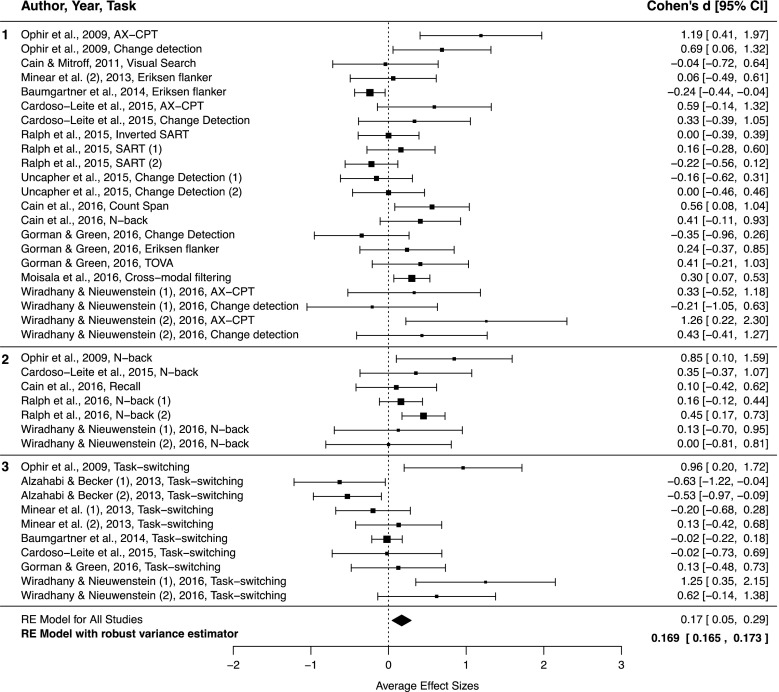
Forest plot of the effect sizes (Cohen’s *d*) for studies included in the meta-analysis. Studies are grouped according to the type of distraction that was involved in the task, with 1 denoting environmental distraction, 2 denoting memory-based distraction, and 3 denoting distraction caused by a previously used task set in a task-switching experiment. *Error bars* represent 95% confidence interval of the effect size. RT =: response times; FA = false alarm rate; CPT = continuous performance task; TOVA = Test of Variables of Attention

Figure 15 shows a forest plot with the effect sizes that were included in the meta-analysis. The effect sizes are grouped by the type of distraction that was involved in the task (environmental, memory based, or task set), and the effects that were found by Ophir et al. (2009) are listed first for each type of distraction. This visualization of effects shows that the majority of studies investigating the association between media multitasking and distractibility link yielded nonsignificant results, as the confidence intervals for the majority of effects included zero. To estimate the mean effect size, we conducted a meta-analysis using a random-effects model. The results of this analysis showed a small but significant, positive association between media multitasking and distractibility, with *d* = .17, 95% CI [.165, .173], *p* = .007, one-tailed. At the same time, however, the analysis also made clear that there was significant heterogeneity amongst the effects in the analysis, *I* ^2^ = 57.02%, *p <* .0001.

#### Moderator analyses

To determine if the heterogeneity of the effects of different studies can be explained in terms of differences between studies examining different types of distractibility, populations of participants, or methods of analyses, we conducted three moderator analyses. These analyses revealed that there were no differences between studies examining different types of distractibility, participants from different populations, or different methods of analysis, with *F*(2, 36) = 1.11, *p* = .342, *F*(2, 36) = .29, *p* = .745, and *F*(2, 36) = 2.81, *p* = .074, respectively.

#### Funnel plot and small-study effects

**Fig. 16:**
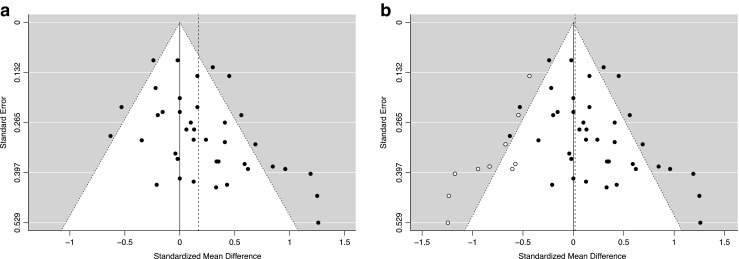
**a** Funnel plot showing the relationship between the effect sizes and standard errors of previous studies into the relationship between media multitasking and distractibility. Effect sizes are plotted along the *x*-axis and standard errors along the *y*-axis, and the g*ray areas* denote the areas in which effects were statistically significant. The *vertical dashed line* indicates the estimated mean effect size. **b** Funnel plot including the effects that were imputed using the trim and fill method

Next, we examined whether the data set showed evidence for small-study effects. To this end, we constructed a funnel plot in which effect sizes are plotted as a function of their standard error, and in which the areas of statistical significance (*p* < .05) were highlighted. In the absence of small-study effects, this plot should form a symmetrical funnel distribution of effect sizes around the mean effect size. As can be seen in Fig. 16a, however, the distribution is clearly asymmetrical, with a preponderance of small sample (large *SE*) studies showing large, positive effects, and a relative lack of similarly imprecise studies showing effects on the other side of the mean effect size. As a formal verification of this impression, we conducted Egger’s test (Egger et al., 1997) to examine the relationship between effect sizes and standard errors. This test showed that this relationship was significant, *Z* = 2.83, *p =* .005, thus underscoring the presence of funnel plot asymmetry.

In interpreting the asymmetrical distribution of small-sample studies, it is important to note that the studies that appear to be missing on the lower left side of the funnel are studies that would be expected to have yielded either nonsignificant or negative results. This observation is indicative of reporting bias, as the asymmetry appears to be associated with the direction and significance of outcomes (Carter & McCullough, 2014; Peters et al., 2007). Furthermore, it also seems unlikely that the asymmetry can be explained in terms of true heterogeneity between studies, as our moderator analyses made clear that this heterogeneity could not be explained in terms of differences between tasks, study populations, or methods of analysis. Accordingly, it seems possible that the reason for the asymmetrical distribution of small studies could be reporting bias, thus warranting further corrective procedures to determine what the estimated effect size would be when this bias is corrected for. To do so, we performed two corrective procedures. First, we used the trim-and-fill procedure to impute the ostensibly missing effects on the left side of the funnel and to recalculate the overall effect size (Duval & Tweedie, 2000). This analysis showed that the association between media multitasking and distractibility turned nonsignificant after correction, with Cohen’s *d* = .07, and *p* = .81 (see Fig. 16b). Secondly, we used a regression-based method that has been deemed more suitable for data sets with relatively high heterogeneity, as is true for the current dataset (Moreno et al., 2009). With this method, we estimated the expected effect size for a study with a standard error of zero. The results of this analysis corroborated the outcome of the trim and fill procedure in that it yielded an effect size of Cohen’s *d* = .001. Taken together, these results make clear that the earlier estimated effect size was strongly influenced by the presence of small-study effects, such that the small but significant association turned nonsignificant after correction for these effects.^4^

## General discussion

In a pioneering study, Ophir et al. (2009) found that people with higher scores on a questionnaire measure of media multitasking show an increased susceptibility to distraction in various laboratory tasks of information processing. While subsequent studies did show associations between media multitasking and various outcome measures other than those used by Ophir et al., they generally failed to replicate the original findings, thus casting doubt on the existence of an association between media multitasking and distractibility. In the current study, we conducted two replication studies to determine the replicability of the original findings by Ophir et al., and we conducted a meta-analysis to assess the existence and strength of the association between media multitasking and distractibility across all studies that compared the performance of HMMs and LMMs on laboratory tests of distractibility in information processing. The results of our replication studies showed only weak and partial support for the findings of Ophir et al., such that only five of our 14 tests yielded a successful replication according NHST, whereas a Bayesian analysis indicated that only two of these effects were based on convincing evidence for an association between media multitasking and distractibility. Furthermore, the results of our meta-analysis showed that the association between media multitasking and distractibility is weak and strongly influenced by small-study effects, such that the application of two corrective procedures for small-study effects changed the estimate of the overall effect size from a significant Cohen’s *d* of .17 to a nonsignificant effect of .01–.07.

Taken together, the results of our work present reason to question the existence of an association between media multitasking, as defined by the MMI or other questionnaire measures, and distractibility in laboratory tasks of information processing. This reason is that our meta-analysis shows that the association between media multitasking and distractibility approximates an effect size of zero after correction for small-study effects. What remains to be explained then is why some studies did show evidence of such an association, including some of the current replication tests. As a case in point, consider the results of the current replication studies. Although the outcomes of these tests generally failed to replicate the effects of Ophir et al. in terms of statistical significance and Bayes factors, the outcomes did consistently show nonsignificant effects in the direction of HMMs being more vulnerable to distraction then LMMs. Accordingly, one may ask how it is possible that so many tests consistently showed a difference in one particular direction, given that this difference does not exist according to the meta-analysis. Importantly, however, this state of affairs might be less telling or mysterious as it seems. To start, it is important to note that our replication attempts were implemented as two independent studies using a between-group comparison in which HMMs and LMMs were compared on seven indices of distractibility. Given that these indices would be expected to be correlated within the same subjects, especially when they derive from the same task, it becomes clear that any coincidental difference in distractibility between the LMM and HMM groups would translate into a consistent pattern across the seven indices. Likewise, when considering the broader literature, it is noteworthy that our meta-analysis makes clear that, regardless of statistical significance, there are 11 studies showing greater distractibility in LMMs, three studies showing no difference between LMMs and HMMs, and 25 studies showing greater distractibility in HMMs (see Table 2). Given that our analysis also suggests the existence of a bias against small-sample studies showing negative and nonsignificant results, it becomes clear that the distribution of studies showing positive and negative results is not so much different than what would be expected for a set studies that tested the outcomes stemming from a distribution that is centered at an effect size of zero.

An alternative interpretation of the current findings might be that the association between media multitasking and distractibility does exist, but that it is very weak. This conclusion would stem from considering the effect size estimate without any correction for small-study effects. Under this interpretation, an important implication of the current work is that future studies into the relationship between the media multitasking and other outcome measures should take into account the fact that these relationships is likely to be very small and only detectable using extremely large samples of participants. To be precise, to achieve 80% power to detect an effect with an effect size of .17 one would need 428 participants per group for the HMM and LMM groups.

In considering whether or not such large-scale studies would show evidence for an association between media multitasking and distractibility in information processing, a last point of note is that perhaps future studies should also use a different calculation of the MMI (see also Baumgartner et al., 2014; Cain et al., 2016). To wit, the current calculation yields a measure of the proportion of media-usage time during which someone uses two media at the same time. This means that a person who spends only 1 hour per day using his laptop while watching television can have the same MMI as a person who does this 16 hours per day. Evidently, if there would exist an association between media multitasking in daily life and performance on laboratory measures of information processing, then this association would be more likely to be seen when using a measure of media multitasking that expresses the amount of time someone spends on this activity (see also Cain et al., 2016; Moisala et al., 2016).

### Conclusions and future directions

The idea that frequent media multitasking could be associated with differences in information-processing capacity is enticing and timely. However, our experiments and meta-analysis did not provide much support for this idea. Instead, our meta-analysis showed that the association between media multitasking and distractibility is likely to be very small, and therefore unlikely to be detected in studies employing relatively small sample sizes. Accordingly a key implication of the current study is that future studies on the link between media multitasking and cognitive functioning should use relatively large samples of participants to ensure sufficient statistical power.

## Electronic supplementary material

ESM 1 (DOCX 63 kb)

## Footnotes

1 The results of these replication tests are presented in the main text, and our analyses for other outcome measures and conditions are reported in a supplementary document.

2 In deciding to include only trials with correct responses to both the first and the last red letter of the sequence, we may have applied an unusually strict criterion for trial inclusion, as previous studies using the AX-CPT typically included trials irrespective of whether the response to the cue was correct. However, since the correct judgment of the last red letter requires a correct judgment of the first, we felt that it was reasonable to use this more strict inclusion criterion. Notably, however, the results did not change when we used the more lenient inclusion criterion of including all trials with a correct response to the last red letter in the sequence.

3 For the study by Cardoso-Leite et al. (2015), we could not include the effect for AX-RT, because these authors only reported an analysis for the average RT on AX and BX trials. Since both types of trials can be assumed to measure the same kind of distractibility effect (cf. Ophir et al., 2009), we included Cardoso-Leite et al.’s effect for average RT in our analysis.

4 It is worth mentioning that we also conducted a meta-analysis using Bayes factors (Rouder & Morey, 2011). This analysis is report in the supplementary document and it yielded an effect size estimate of .03, with strong evidence in favor of the null hypothesis.
