# Supplementary material for: Cognitive control in media multitaskers: Two replication studies and a meta-Analysis
Source: Atten Percept Psychophys. 2017 Aug 24;79(8):2620–41. doi: 10.3758/s13414-017-1408-4 (PMC5662702; doi:10.3758/s13414-017-1408-4)
Supplement: Supplementary file 1 — (DOCX 63 kb) [file 13414_2017_1408_MOESM1_ESM.docx]

**Supplementary materials for Cognitive Control in Media Multitaskers: A Replication Study and Meta-Analysis**

**Experiment 1**

*Change Detection Task: Results for 4 and 6-Target Conditions.* In addition to examining the effects of distraction in the condition with a memory set size of 2 items, we also moved beyond the analyses reported by Ophir et al. (2009) in examining the effects of distractors for the conditions with a memory set size of 4 and 6 items. For the condition with a memory set size of 4 items, there were no main effects of Group *F*(1, 20) = 0.77, *p* = .395, *η^2^* = .024 nor of Distractor Set Size (0, 2, or 4), *F*(2, 40) = .42, *p* = .658, *η^2^* = .006, but we did find a significant interaction of these factors, *F*(2, 40) = 3.98, *p* = .027, *η^2^* = .060. As can be seen in Figure 1a, this interaction effect appeared to derive from the effect that LMMs – not HMMs – were more strongly affected by the number of distractors.


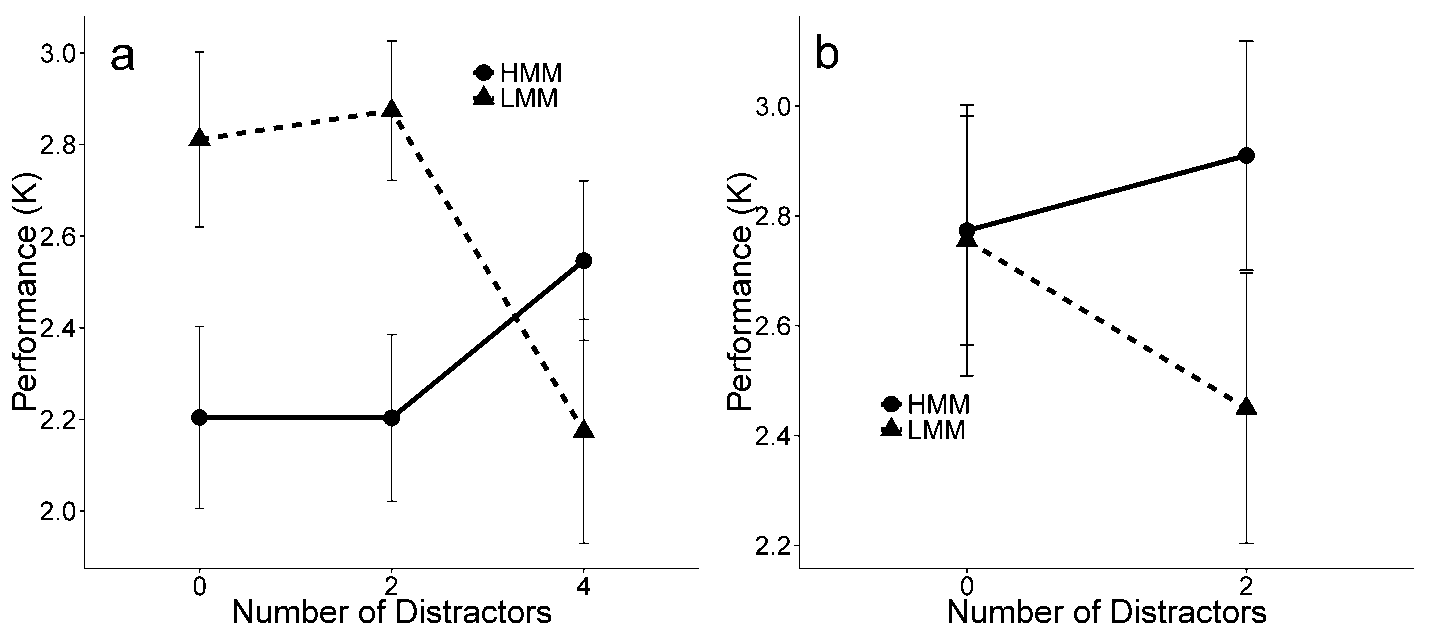


**Figure 1.** Change detection performance for the condition with 4 targets and 0, 2, or 4 distractors (Figure 1a) and the condition with 6 targets and 0 or 2 distractors (Figure 1b) in Experiment 1. Error bars represent within-subjects standard errors of the means (Morey, 2008).

For the condition with a memory set size of 6 items, the analysis showed no main effects of Group *F*(1, 20) = 0.27, *p* = .610, *η^2^* = .009, nor of Distractor Set Size (0 or 2), *F*(1, 20) = .08, *p* = .771, *η^2^* = .001, and the interaction of these factors also failed to reach significance, *F*(1, 20) = .59, *p* = .449, *η^2^* = .008 (Figure 1b).


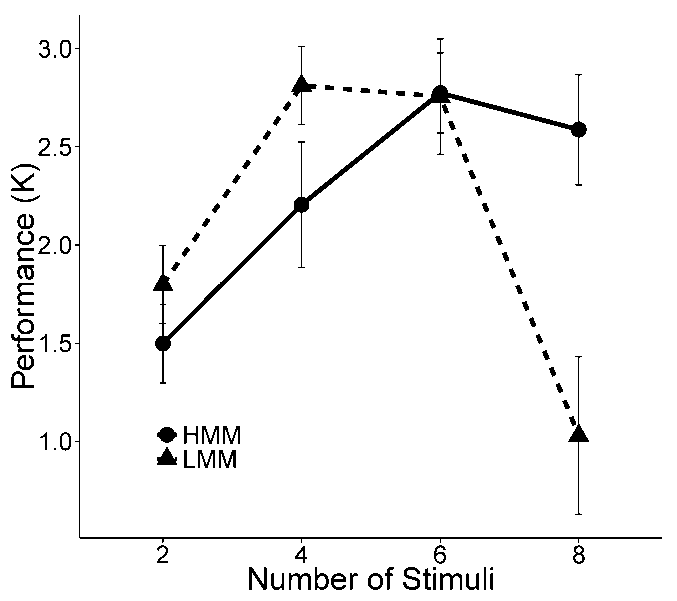


**Figure 2.** Change detection performance for the condition with 2, 4, 6, or 8 targets shown without distractors in Experiment 1. Error bars represent within-subjects standard errors of the means (Morey, 2008).

*Change Detection Task: Results for Conditions without Distractors.* Lastly, we also examined if the change detection task showed any difference in performance between LMMs and HMMs for the conditions without distractors. A repeated measures ANOVA with Group (HMM vs. LMM) and Memory Set Size (2, 4, 6, or 8 items) as factors showed no significant effect of Group, *F*(1, 20) = 0.35, *p* = .561, *η^2^* = .01 but it did show a significant effect of Memory Set Size, *F*(3, 60) = 7.59, *p* < .001, *η^2^* = .18. In addition, the interaction between group and memory set size was significant, *F*(3, 60) = 6.04, *p* = .001, *η^2^* = .15. As can be seen in Figure 2, this interaction was driven by an unexpected drop in performance for the LMMs as set size increased from 6 to 8 items. Further scrutiny of the data obtained in the set-size 8 condition did not yield insight into the reasons for this unexpected drop in performance.

*AX-CPT with Distractors: Results.* There were no significant differences in accuracy between LMMs and HMMs in the AX, BX, AY, and BY trials, all *p*’s > .17. Similarly, the analysis showed no overall difference in response times between HMMs and LMMs, *t*(21) = 1.68, *p =* .054. Further analyses examining response times for the remaining two types of trials – namely those in which the red letters comprised the pair A-Y or B-Y – showed that HMMs were significantly slower than LMMs to respond to BY trials, *t*(21) = 3.12, *p* = .004, but not to AY trials, *t*(21) = .57, *p* = .58, (see Figure 8).

*N-Back task: Results.* Unlike the study by Ophir et al., our analysis of *d’* as a function of Group (LMM vs. HMM) and WM Load (2-back vs. 3-back) did show a significant main effect of WM Load, *F*(1, 21) = 30.95, *p* < .001, *η*^2^ = .361, but no main effect of Group, *F*(1, 21) = 0.50, *p* = .486, *η*^2^ =.015, *d =* .25, and no significant Group × WM Load interaction, *F* (1, 21) = .077, *p* = .783, *η^2^*=.001, *d =* .06. Furthermore, a general linear model of false alarm in the three-back task with Group and Time on Task as predictors showed no main effect of Group, *χ*^2^(1) = 2.4, *p* = .12, no main effect of Time on Task, *χ*^2^(1) = .03, *p* = .86, and no Group × Time on Task interaction, *χ*^2^(1) = 1.9, *p* = .17.

*Task-switching: Results.* There was no significant difference in accuracy between groups on switch or repeat trials, both *p’*s > .116.

**Experiment 2**

*Change Detection Task: Results for 4 and 6-Target Conditions.* For the condition with a memory set size of 4 items (Figure 3a), we found no main effect of Group, *F*(1, 27) = 0.12, *p* = .729, *η^2^* = .002 or distractor set size, *F*(2, 54) = 1.25, *p* = .293, *η^2^* = .02, and the Group × Distractor Set Size interaction also failed to reach significance, *F*(2, 54) = 1.39, *p* = .256, *η^2^* = .02. For the condition with a memory set size of 6 items (Figure 3b), we also found no main effect of Group, *F*(1, 27) < .001, *p* = .983, *η^2^* < .001 or Distractor Set Size, *F*(1, 27) = 3.35, *p* = .078, *η^2^* = .03, and the Group × Distractor Set Size interaction also failed to reach significance, *F*(1, 27) = 2.67, *p* = .114, *η^2^* = .02.

###


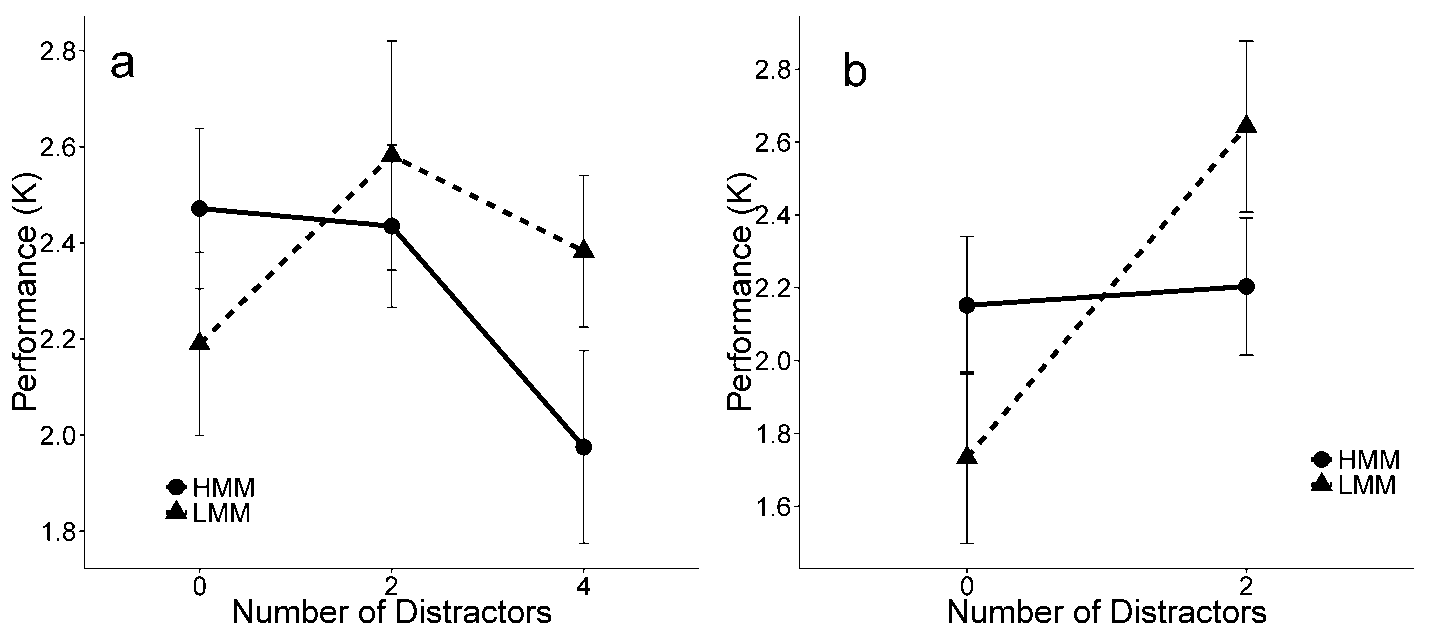


**Figure 3.** Change detection performance for the condition with 4 targets and 0, 2, or 4 distractors (Figure 3a) and the condition with 6 targets and 0 or 2 distractors (Figure 3b) in Experiment 2. Error bars represent within-subjects standard errors of the means (Morey, 2008).

*Change Detection Task: Results for Conditions without Distractors.* A repeated measures ANOVA with Group (HMM vs. LMM) and Memory Set Size (2, 4, 6, or 8 items, without distractors) as factors yielded no main effect of Group, *F*(1, 27) = 0.002, *p* = .969, *η^2^* < .001, no main effect of Memory Set Size, *F*(3, 81) = 2.49, *p* = .065, *η^2^* = .04, and no Group × Memory Set Size interaction, *F* (3, 81) = 1.02, *p* = .387, *η^2^* = .019.


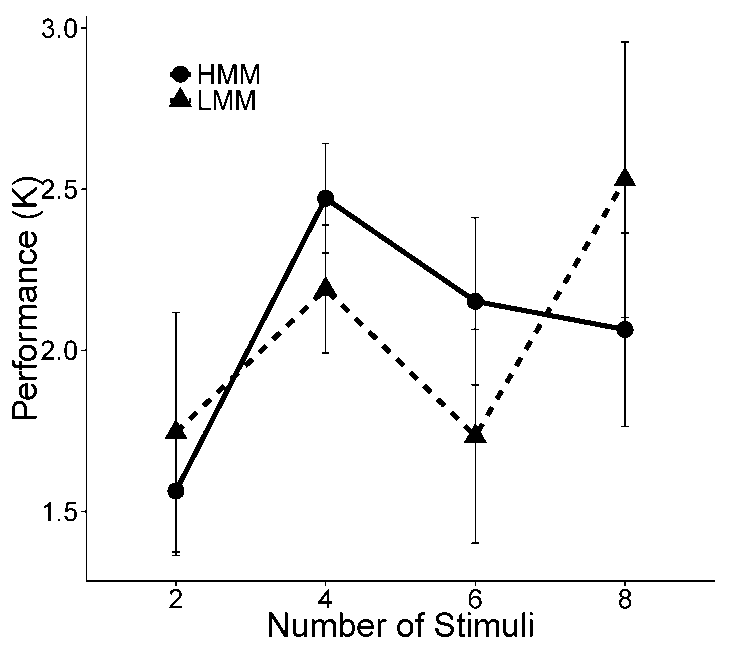


**Figure 4.** Change detection performance for the condition with 2, 4, 6, or 8 targets shown without distractors in Experiment 2. Error bars represent within-subjects standard errors of the means (Morey, 2008).

*AX-CPT with Distractors: Results.* HMMs were more accurate than LMMs in AY trials, t(18) = 2.07, *p =* .05 and LMMs were more accurate than HMMs in BX trials, *t*(18) = - 2.48, *p* = .02. There were no significant differences between the HMM and LMM groups in accuracy for AX and BY trials, *p’*s > .219. Response times also did not differ between groups for the AY, *t*(18) = -1.27, *p* = .221, *d* = -.62, and BY *t*(18) = 1.35, *p* = .192, *d* = .66 trials, respectively.

*N-Back task: Results.* Our analysis of *d’* as function of Group (LMM vs. HMM) and WM Load (2-back vs. 3-back) showed a significant main effect of WM Load, *F*(1, 24) = 13.88,  *p* < .01, *η^2^* = .124, *d =* .75 but no main effect of Group, *F*(1, 24) *=* .066, *p =* .799, *η^2^* = .002, *d =* .09, and no significant Group × WM Load interaction, *F*(1, 24) = .004, *p* = .948, *η*^2^<.001, *d <* .01. Our general linear model of false alarm in the three-back task with Group and Time on Task as predictors showed no main effect of Group, *χ*^2^(1) = 1.4, *p* = .24, no main effect of Time on Task, *χ*^2^(1) = .041, *p* = .84, and no Group × Time on Task interaction, *χ*^2^(1) = 1.6, *p* = .2.

*Task-switching: Results.* There is no difference in accuracy between HMMs and LMMs for repeat and switch trials, all *p*’s > .340.

**Meta-analytic Bayes Factor**

In a meta-analytic Bayes Factor, we assumed a true effect size δ which is constant across experiments and a varying variance (Rouder & Morey, 2011). If the null hypothesis is true, the posterior distribution of effect sizes would be peaked closer to zero. On the other hand, if the alternative hypothesis is true, the posterior distribution of effect sizes would be peaked further away from 0. Under these assumptions, a Bayes Factor was calculated using the meta.ttestBF function of the BayesFactor package in R. The prior distribution of the effect size was set to $\sqrt{2}$/2 (default) and one-sided analysis was conducted assuming that HMMs performed worse than LMMs in all cases. In cases of effect sizes which were not computed from *t-tests*, the *t* values were estimated using the formula below (Borenstein, Hedges, Higgins, & Rothstein, 2009)

$$t= \frac{d\sqrt{{(n}_{1}+n_{2}-2) (n_{1}n_{2})}}{n_{1}+n_{2}}$$

Where *d* is the estimated *Cohen’s d* from the previous calculation and n_1_ and n_2_ are the sample size of HMMs and LMMs, respectively.


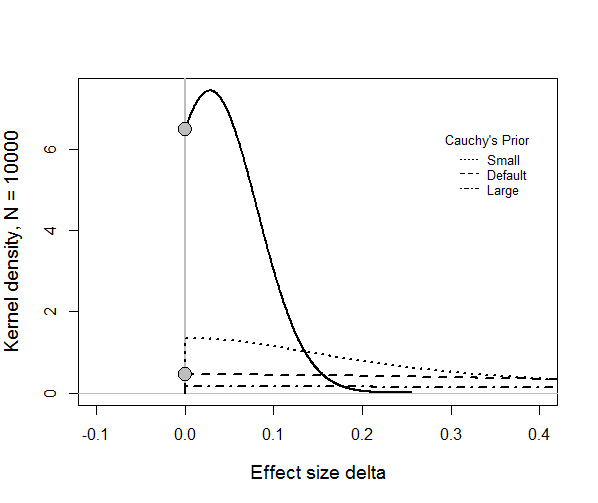


Figure 5. Density simulation of effect size δ of the association between media multitasking and increased distractibility with N = 10000 iterations. The dotted, dashed, and dash-dot lines show skeptical, default, and optimistic Cauchy priors, respectively.

Figure 5 shows the posterior effect size of the overall experiment. The density distribution peaked at an effect size δ = .03, indicating that the most probable effect size estimation lies around .03. This effect size has a Bayes Factor of 8.50 in favor of the null hypothesis, indicating that it is approximately 8 times more likely that there is no association between media multitasking and increased distractibility.

To explore if this finding was not due to our selection of the prior distribution, we performed a sensitivity analysis (Kass & Raftery, 1995). That is, we evaluate Bayes Factor over a range of prior possibilities. In this case, the priors were varied from small (1/3 of the default prior) to large (3 × the default prior). The small prior peaked closer to 0, indicating a skeptical prior while the large prior has a wider distribution on the negative effect size, indicating an optimistic prior (see Figure 5). In both cases, the data support the null hypothesis, with *BF*_01_ = 2.88 and *BF*_01_ = 25.45 for the small and large priors, respectively, meaning that it is 3 to 25 times more likely that there is no association between media multitasking and increased distractibility.

Additional References

Borenstein, M., Hedges, L. V, Higgins, J. P. T., & Rothstein, H. R. (2009). *Introduction to Meta-Analysis*. West Sussex: John Wiley & Sons.

Kass, R. E., & Raftery, A. E. (1995). Bayes Factors. *Journal of the American Statistical Association*, *90*(430), 773–795.

Morey, R. D. (2008). Confidence Intervals from Normalized Data: A correction to Cousineau ( 2005 ). *Tutorials in Quantitative Methods for Psychology*, *4*(2), 61–64. http://doi.org/10.3758/s13414-012-0291-2

Rouder, J. N., & Morey, R. D. (2011). A Bayes factor meta-analysis of Bem’s ESP claim. *Psychonomic Bulletin & Review*, *18*(4), 682–689. http://doi.org/10.3758/s13423-011-0088-7
